# *Malassezia* in environmental studies is derived from human inputs

**DOI:** 10.1128/mbio.01142-25

**Published:** 2025-05-19

**Authors:** Saleh Rahimlou, Anthony S. Amend, Timothy Y. James

**Affiliations:** 1 Department of Ecology and Evolutionary Biology, University of Michigan 1259, Ann Arbor, Michigan, USA; 2 Pacific Biosciences Research Center, University of Hawaii at Mānoa, Honolulu, Hawaiʻi, USA; University of California, Berkeley, Berkeley, California, USA

**Keywords:** dandruff, dermatitis, lipid, mycobiome, skin, yeast

## Abstract

The fungus *Malassezia* is the most prevalent member of the human skin mycobiota, known for its lipid dependence and inability to synthesize long-chain fatty acids. *Malassezia* exhibits remarkable niche breadth, inhabiting soils, dust, as well as various marine environments from polar regions to deep-sea vents. The consistent presence of *Malassezia* in marine habitats, including those associated with marine animals, suggests that the fungus is able to grow and play a fundamental role in marine ecosystems. In this study, we explored metagenomic sequences from the Sequence Read Archive database to investigate the presence, distribution, and origins of *Malassezia* species within marine ecosystems, with the goal of assembling *Malassezia* genomes. Using various search methods, we found that up to 10% of the shotgun and amplicon data sets analyzed contained *Malassezia* sequences. However, the relative abundance of *Malassezia* in each data set was low, and there was a strong correlation between human and *Malassezia* sequences in marine environments, consistent with contamination. From the most *Malassezia*-rich data sets, we attempted to generate metagenome-assembled genomes. However, *Malassezia* contigs together showed low genome completeness levels, with a maximum of 2.2% in a sample. Phylogenetic analysis identified the contigs as closely related to human-associated *Malassezia globosa* and *Malassezia restricta*, along with *Malassezia sympodialis* and *Malassezia pachydermatis*, which were the most prevalent species across all studied environments. Our data suggest that many observations of *Malassezia* in the environment are derived from human sources and that widespread contamination confounds the exploration of *Malassezia* diversity and ecological role in the marine environment.

**IMPORTANCE:**

*Malassezia* is the singular fungus most associated with humans. It colonizes mammalian skin and requires host-derived fatty acids to grow. Widespread sequencing of environmental DNA surprisingly showed that *Malassezia* is also ubiquitous outside of mammalian hosts. *Malassezia* is frequently found in marine habitats where it is associated with corals, deep sea vents, diatoms, and more. Given its widespread presence, we reasoned that public metagenomic data could be used to assemble a genome sequence of an uncultured marine *Malassezia*. However, we found that *Malassezia* was ubiquitous but never abundant in marine samples and that the few metagenomes we could assemble were consistent with recent human introduction. We also found that the presence of human DNA in sequencing data sets is strongly correlated with the presence of *Malassezia* DNA, and while not ruling out the growth and survival of *Malassezia* in marine habitats, they suggest widespread contamination of public data with *Malassezia*.

## INTRODUCTION

*Malassezia* spp. are the most prevalent members of the human skin mycobiota (1) and are also key components of the animal skin mycobiota, where they function as both commensal and opportunistic pathogens (2). They are unable to synthesize long-chain fatty acids due to their lipid dependence (except *Malassezia pachydermatis*) and, therefore, have to rely on external sources, which makes them difficult to cultivate (3). *Malassezia* species thrive in areas of the skin that are rich in sebaceous glands, such as the scalp, face, and upper trunk. While not usually harmful, *Malassezia* is considered an opportunistic pathogen and is associated with several common skin diseases in humans, including pityriasis versicolor, seborrheic dermatitis, and dandruff (4). *Malassezia* inhabits not only the skin but also the human gut and is common in hospital environments (2). *Malassezia* also have beneficial roles, such as lipid metabolism and the decomposition of skin oils, and may play a crucial role in maintaining skin homeostasis through its interactions with the immune system and other microbes (5).

The genus *Malassezia* consists of 18 species and belongs to the subphylum Ustilaginomycotina within the Basidiomycota (6). Fungi in Ustilaginomycotina are primarily known for their pathogenic relationships with plants, particularly grasses. They infect various cereal crops, causing diseases such as smut and bunt. *Malassezia* species are divided into three groups, with one group further subdivided. Group A species are robust in culture, frequently used as models for the genus, and often isolated from septic infections and hospital outbreaks. Despite being easy to culture, *Malassezia furfur* from Group A is rarely detected on healthy human skin by molecular methods, likely due to differences in growth rates. Group B1 species like *Malassezia pachydermatis* are primarily found on animal skin and linked to clinical outbreaks, while *Malassezia restricta* and *Malassezia globosa* are common on human skin but difficult to culture. Group B2’s *Malassezia sympodialis* is more common in colder climates and is associated with inflammatory skin conditions. Group C includes more divergent species like *Malassezia slooffiae*, *Malassezia cuniculi*, and *Malassezia vespertilionis*, found in various mammals (2, 7).

It is believed that *Malassezia* exhibits a remarkable ecological niche breadth, being detected via metabarcoding in various marine environments ranging from polar regions to deep-sea vents (8–10). These data suggest that many *Malassezia* species discovered in marine environments are also present in terrestrial ecosystems. In addition, *Malassezia* populations from both land and sea exhibit significant intermingling within and across clades (10). This suggests that there was not a singular evolutionary event leading to the divergence of terrestrial and marine *Malassezia* lineages, as distinct marine and terrestrial clades would have emerged if such an event had occurred. However, the question of how free-living *Malassezia* species found in various aquatic environments, such as rock swabs, water samples, and sediments, procure the lipids they need remains a subject of debate and inquiry.

A survey of the literature indicates that *Malassezia* has been detected in high-throughput sequencing data across a diverse array of habitats, including soil, sediments, dust, plants, animals, and various other environmental contexts (e.g*.*, references 11–16). We also looked at the GlobalFungi database and found that out of 84,000 amplicon data sets, almost 12,000 (ca. 14%) contained *Malassezia* from various environments (17). The wide distribution of *Malassezia* across diverse habitats raises questions about its adaptability, ecological versatility, and survival strategies in different environmental conditions. Given the widespread presence of *Malassezia* sequences across various habitats, its dependence on lipids, and the uncertainty of whether environmental sequence types are distinct from those associated with humans, there exists the possibility of widespread contamination of samples from humans.

Microbiome studies are highly susceptible to contamination from multiple sources, often making it difficult to trace the origin of contaminant DNA (18). Contamination can occur during sampling due to environmental exposure or mishandling during collection, transport, and shipment. Additionally, laboratory procedures pose a significant risk. Improper aseptic techniques and suboptimal laboratory workflows can exacerbate contamination issues. Another major concern in fungal microbiome research is the presence of trace fungal DNA in commercially available PCR reagents, including lyophilized primers, TaqMan probes, and master mix solutions (19). *Malassezia* and several other fungal contaminants have been identified in laboratory reagents where they were likely introduced by the skin of scientists (19). Such contamination can lead to false-positive results in PCR assays, particularly in low-biomass samples where even minimal background contamination can obscure true fungal detection. This issue affects both targeted PCR-based sequencing and broader metagenomic approaches. Sequencing technologies also introduce additional risks of false-positive signals due to technical limitations such as cross-contamination and index bleed-through (20, 21). Furthermore, bioinformatic analyses can contribute to false-positive results due to sequence misidentification, arising from various computational errors (22).

Proper estimation of which fungi are actually key components of the marine mycobiota is critical for understanding ecosystem function and stability. Fungi are a critical but underexplored component of marine ecosystems, playing key roles in nutrient cycling, organic matter decomposition, and symbiosis with other marine organisms (23). Given the significance of marine ecosystems for planetary health, there has been an increasing number of environmental DNA studies focused on these ecosystems. One of the largest expeditions is the *Tara* Oceans project, which sampled 210 global oceanic stations to catalog biodiversity (24). Recent studies utilizing the extensive *Tara* Oceans data set have shed light on the diversity and distribution of marine fungi, including the widespread prevalence of *Malassezia* species (25). Strikingly, in this study using 18S ribosomal DNA metabarcoding, a *Malassezia* operational taxonomic unit (OTU) was the fifth most abundant among fungi detected and occurred in the highest proportion of samples (93% of all samples). Moreover, several studies detecting *Malassezia* RNA transcripts in deep ocean sediments, as well as in association with corals and phytoplankton, suggest that *Malassezia* may be present and active in marine ecosystems (26–28).

In this study, we analyzed metagenomic sequences from the Sequence Read Archive (SRA) across a variety of environments to explore the presence, distribution, and potential origins of *Malassezia* species in marine ecosystems. We hypothesized that if *Malassezia* is capable of thriving in marine environments and is not merely a contaminant, its genomes could be assembled from marine metagenomic data and subsequently compared to those of human-associated strains. Additionally, considering the influence of anthropogenic factors, we assumed that *Malassezia* would be more abundant in coastal regions than in offshore, open ocean environments, where human-associated microbial contaminants are less common. By identifying distribution patterns of *Malassezia* across various ecosystems, this study aims to provide a deeper understanding of how potential organisms can interfere with and obscure the results of microbiome studies.

## MATERIALS AND METHODS

### Leveraging indexed databases: BigQuery and PebbleScout

The Sequence Read Archive uses the NCBI SRA Taxonomy Analysis Tool (STAT) to calculate the taxonomic distribution of reads from next-generation sequencing runs. STAT maps sequencing reads to a taxonomic hierarchy using exact matches between query reads and precomputed k-mer dictionary databases (29). STAT combines taxonomic information with metadata to offer comprehensive insights to users. The SRA has deposited its metadata into BigQuery (Google Cloud Platform) to provide the bioinformatics community with programmatic access to this data. Users can search across the entire SRA by sequencing methodologies and sample attributes. In order to understand factors that can drive the occurrence of *Malassezia* in environmental samples, we looked for the presence of co-occurring taxa in SRA data sets. We utilized SQL queries via BigQuery to search the NCBI SRA datastore for occurrences of both *Malassezia* and Hominidae hits on 11,510 metagenomic data sets sampled from marine environments. Considering Hominidae as the primary host for *Malassezia*, the correlation between these two entities can serve as an indicator of potential contamination. In addition, to broaden our analysis across various habitats, we utilized BigQuery to retrieve metagenomic data spanning diverse organisms and environments (library source = metagenomic). We filtered out data originating from human sources and determined the correlation between the occurrences of *Malassezia* and Hominidae hits. To enhance the clarity of the relationships between the variables, we log transformed the read counts of both *Malassezia* and Hominidae. Additionally, we assessed the correlation between the occurrence of other fungi known to be highly contaminating in laboratory settings and Hominidae. We calculated the Pearson correlation coefficient on log-transformed values using the statistical software R and visualized these using the ggplot2 package (30). Additionally, we calculated the Pearson correlation coefficient for other fungi and bacteria, employing the same methodology. This approach was implemented to eliminate the possibility of any spurious correlations, ensuring the reliability of our findings.

We also employed BigQuery to identify data sets exhibiting higher levels of *Malassezia* abundance within marine environments. To achieve this, we specified our search criteria to include data sets categorized under the organism “marine metagenome” and sourced from “metagenomic” libraries. This focused approach allowed us to pinpoint and analyze metagenomic data sets that are rich in *Malassezia* specifically within marine ecological settings. To explore the presence of *Malassezia* across diverse habitats such as dust, human, marine, plant, rhizosphere, root, and soil, we employed BigQuery to quantify the *Malassezia* hits in all available data from these environments up to 8 February 2024, encompassing all sequencing approaches. We then applied a linear regression model across diverse habitats from which metagenomic samples were obtained. We utilized the ggplot2 package to create visual representations of the number of hits observed across different habitats in our analysis. In addition, we utilized PebbleScout (https://pebblescout.ncbi.nlm.nih.gov) using ITS sequences from 16 *Malassezia* species as queries and selecting “metagenomic” and “WGS” databases. PebbleScout is a tool designed to efficiently navigate large nucleotide databases, such as the Sequence Read Archive and GenBank, by indexing sequences for quick searching (31). It enables researchers to find relevant runs or assemblies based on short sequence matches to user queries, ranking results by their informativeness, thus simplifying the process of data analysis in vast genomic resources.

### Exploration of metagenomic data in marine environments

**Fig 1:**
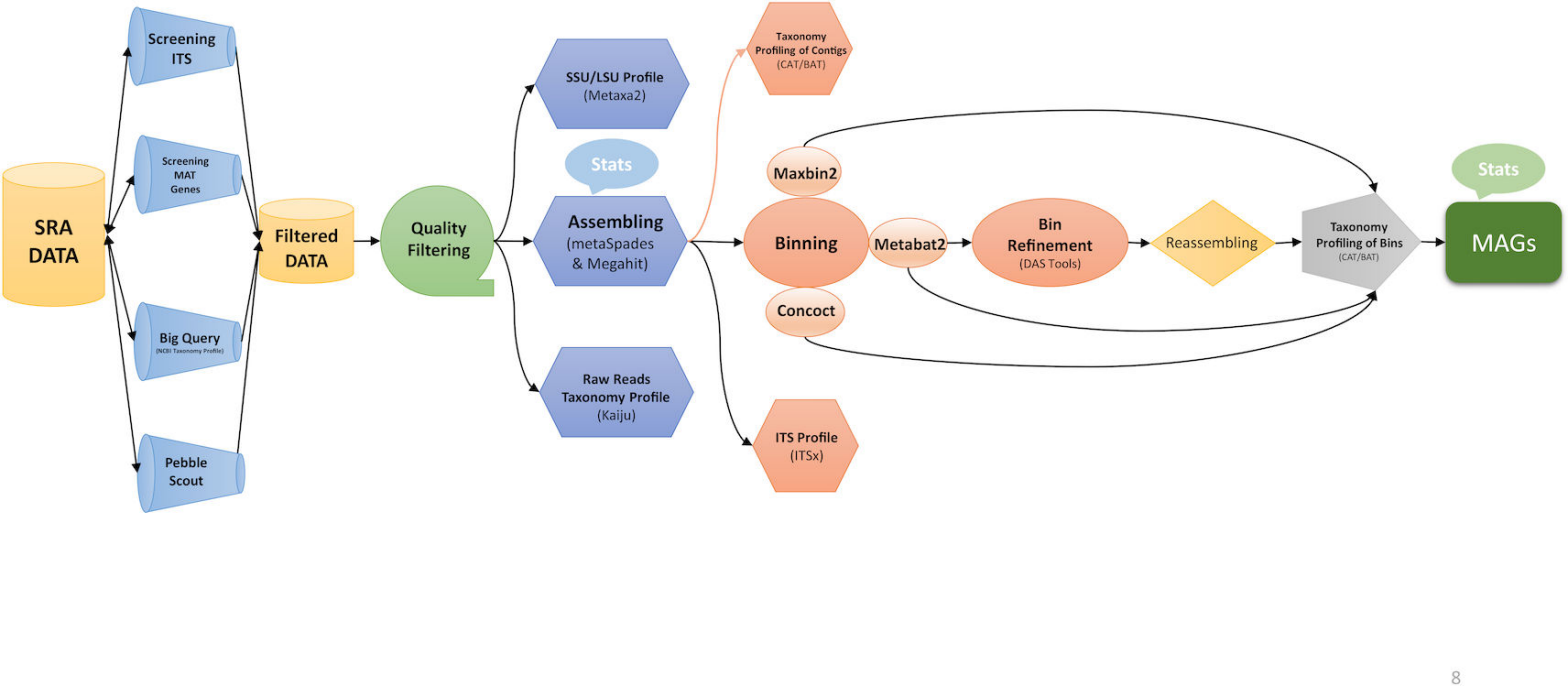
MAG-Explorer is an automated pipeline developed in Nextflow version 24.04.1 to search and extract metagenome-assembled genomes from SRA data sets.

A custom pipeline (MAG-Explorer) was utilized to scan through 11,510 metagenomic data sets from marine environments, searching for sequences of ITS and mating type (MAT) genes from all 16 *Malassezia* species. ITSx (32) was used to extract the ITS1 and ITS2 spacer regions from each query sequence. Then, bwa mem (33) with parameters L 100 -T 32 was used to map reads to queries. Data with higher *Malassezia* abundance were subjected to our MAG-Explorer pipeline for *Malassezia* genome extraction (Fig. 1). The MAG-Explorer pipeline is crafted using the Nextflow DSL2 language (34). This advanced scripting language facilitates the creation of scalable and reproducible workflows. MAG-Explorer starts by assembling reads with metaSPAdes version 3.15.5 (35) using various k-mer sets (21, 45, 65, 85, and 105). The contigs are then grouped into taxonomic bins using Metabat2 (36), Maxbin2 (37), and Concoct (38). The bins are refined into a non-redundant set using DASTool version 1.1.6 (39). MetaWrap version 1.3.2 (40) is then employed to reassemble the bins to enhance the contiguity of the assembly, and CAT/BAT version 5.2.3 (41) is used to assign taxonomy to the bins. CAT is then utilized to assign taxonomy to each contig in the assembly as well, ensuring comprehensive tracking of taxa. Additionally, Kaiju version 1.10.1 (42) is employed to assign taxonomy to sequencing reads prior to assembly, enhancing the accuracy and completeness of taxonomic assignments. Metaxa version 2 (43) and ITSx version 1.1.3 (32) are employed to extract SSU/LSU and ITS sequences, either before or after assembly, respectively. Busco version 5.1.12 (44) is then used to measure the completeness of the bins identified as *Malassezia* using the “fungi_odb10” lineage.

To compare genomes, MUMmer version 4.0.0 (45) was utilized to align the *Malassezia* contigs recovered from the SRA data sets to the existing *Malassezia* genomes, employing the default parameter settings. To place marine data sets containing *Malassezia* onto a map of the world and estimate distance from the shore, the BioSample accessions for SRA sequencing run accessions from marine environments were retrieved, and Esearch version 16.2 from the Entrez direct package (46) was utilized to obtain the coordinates for samples deposited in GenBank. Out of 11,510 accessions, it was found that 572 were missing coordinates, had incorrect coordinates, or had coordinates that matched land instead of marine environments. After removing these samples, the coordinates were used to plot the abundance of *Malassezia* reads for each BioSample accession on a world map using the ggplot2 package. Additionally, the nearest distance to land was calculated for each coordinate using a custom R script to assess whether *Malassezia* abundance diminishes with increasing distance from the coastline.

### Exploration of amplicon data

We utilized a custom Perl script to scan through 188,409 fungal ITS amplicon data sets, searching for ITS1 and ITS2 sequences from 16 *Malassezia* species. We retrieved metadata linked to those data sets using Esearch version 16.2 from the Entrez direct package (46). The reads were quality trimmed using Seqtk (https://github.com/lh3/seqtk) and then aligned to the query ITS spacer regions using BBMap (sourceforge.net/projects/bbmap). Hits were then tallied for each query sequence. We plotted the frequency of hits for various *Malassezia* species from different habitats using the ggplot2 package in R.

### Phylogenetic tree construction

The metagenomic bins identified as *Malassezia* were subjected to gene prediction. Initially, gene prediction was conducted using Augustus version 3.5.0 (47) with training based on *Ustilago maydis* species. A custom Python script was then employed to extract the predicted genes from all *Malassezia* genome assemblies. Subsequently, the extracted genes underwent alignment using MAFFT version 7.520 (48) and trimming using TrimAl version 1.4.1 (49) with a 0.1 gap penalty. The resulting nucleotide alignment matrix was utilized for tree construction using IQtree version 2 (50), employing the ModelFinder Plus option to select the optimal model of sequence evolution with incorporating 100 bootstrap replicates. Finally, the constructed tree was visualized using FigTree version 1.4.3 (http://tree.bio.ed.ac.uk/software/figtree). The pipeline designed for the construction of a phylogenetic tree using partial genomes is available in the GitHub repository.

## RESULTS

**Fig 2:**
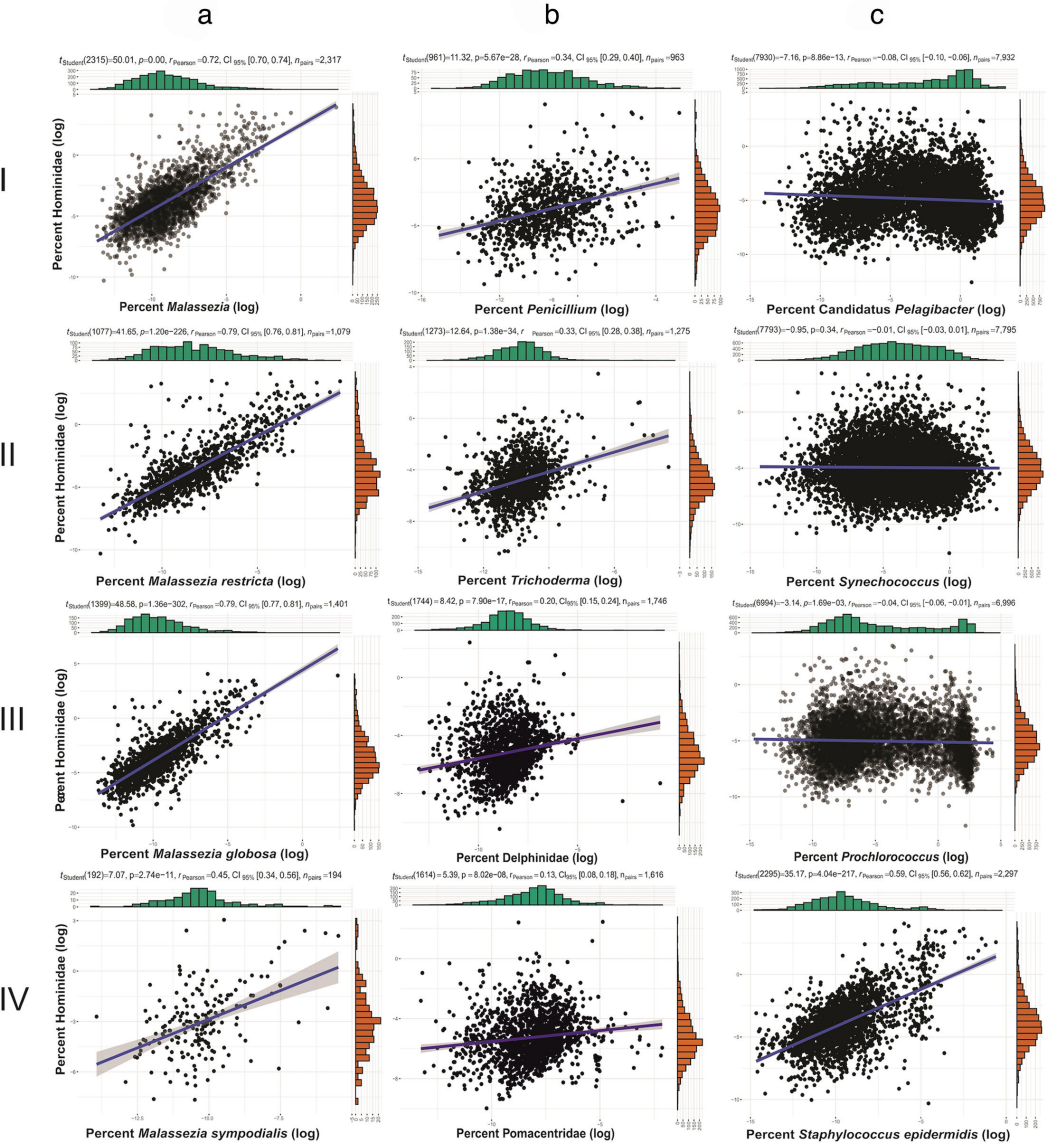
Correlation analysis of log-transformed and depth-corrected (percent) hits for Hominidae and *Malassezia* within marine shotgun data extracted using BigQuery. Panel a shows the correlation between *Malassezia* and Hominidae hits. Panel b(I,II) illustrates the correlation between common fungal contaminants and Hominidae. Panel b depicts the correlation between marine-exclusive bacteria and Hominidae. Panel c(IV) represents the correlation between the human-associated bacterium *Staphylococcus epidermidis* and Hominidae.

In our investigation using BigQuery, we analyzed 11,510 metagenomic data sets from marine environments. Among these, 2,358 (20.5%) data sets contained *Malassezia* sequences. Notably, the vast majority (98.3%) of data sets with *Malassezia* sequences also contain human sequences (*n* = 2,318), while 8,556 data sets (74.3%) were found to contain human (Hominidae) sequences, meaning most marine metagenomic data sets have human DNA in them. We observed a significant correlation between the number of human and *Malassezia* sequences (*P* = 0.00, *R*_Pearson_ = 0.72; Fig. 2). We then expanded our approach and conducted the same analysis on SRA data sets from diverse environments. Examining shotgun data sets from diverse origins, we observed a strong correlation between *Malassezia* and Hominidae hits (*P* = 0.00, *R*_Pearson_ = 0.61; Fig. S1). To confirm that the human sequences are indeed of human origin, we mapped the raw sequencing reads to the GRCh38 human reference genome assembly and calculated the percentage of reads that mapped. The results were consistent with the percentages reported by GenBank generated via the STAT tool (Table S1). The measured correlations between widespread fungal contaminants and Hominidae sequences were also positive, though weaker than those for *Malassezia* (Fig. 2). We also observed a weak but positive correlation between Hominidae and other eukaryotes, such as Delphinidae, the closest marine lineage to humans (Fig. 2), and marine fish Pomacentridae (Fig. 2). In contrast, there was nearly zero correlation with marine-exclusive bacteria, including Candidatus *Pelagibacter*, *Synechococcus*, and *Prochlorococcus* (Fig. 2). However, we observed a strong correlation between *Staphylococcus epidermidis*, a member of the normal human microbiota, and Hominidae sequences (*P* = 4.05e−217, *R*_Pearson_ = 0.59; Fig. 2).

**Fig 3:**
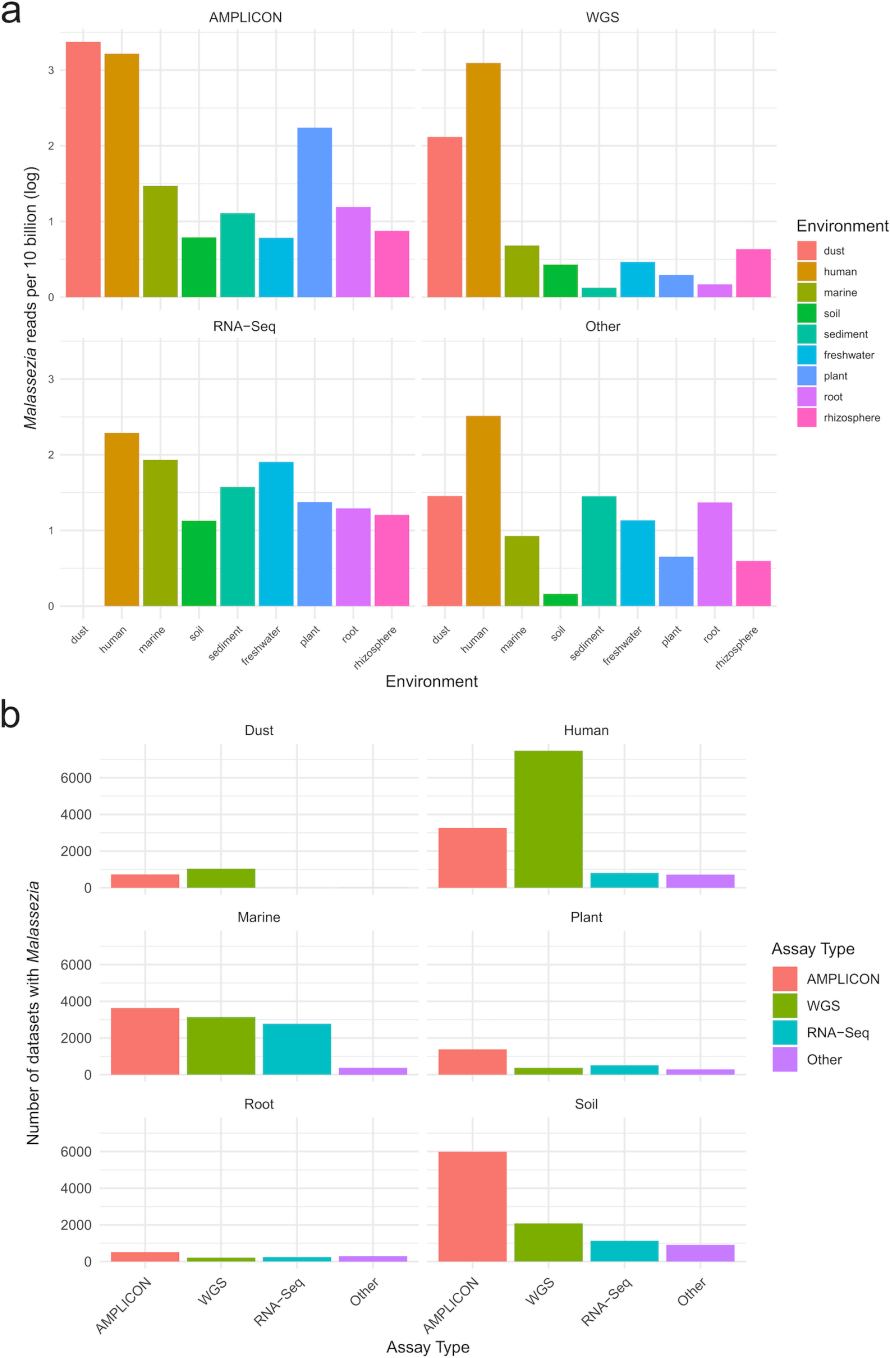
The abundance of *Malassezia* sequences (log) in 10 billion shotgun sequences generated in different environmental settings, calculated using BigQuery (a) Number of data sets with at least one sequence matching *Malassezia* across different sequencing approaches in various environments (b).

These strong correlations of *Malassezia* with human DNA are suggestive of potential widespread co-introduction of human and *Malassezia* DNA across nearly all environments studied. We also utilized BigQuery to scour approximately 1 billion data sets in the SRA database, examining data from diverse habitats, including dust, humans, soil, roots, rhizosphere, and plants. *Malassezia* was detected in all types of non-marine habitats, across various assay types. Notably, dust and human samples exhibited a significantly higher abundance of *Malassezia* reads (*P* < 0.05; Fig. 3). We found that *Malassezia* was underrepresented in amplicon data sets and overrepresented in whole genome shotgun and particularly RNA-seq data sets. This bias may occur due to the amplicon being skewed heavily toward bacterial 16S RNA data sets and RNA-seq biased toward eukaryotes.

**Fig 4:**
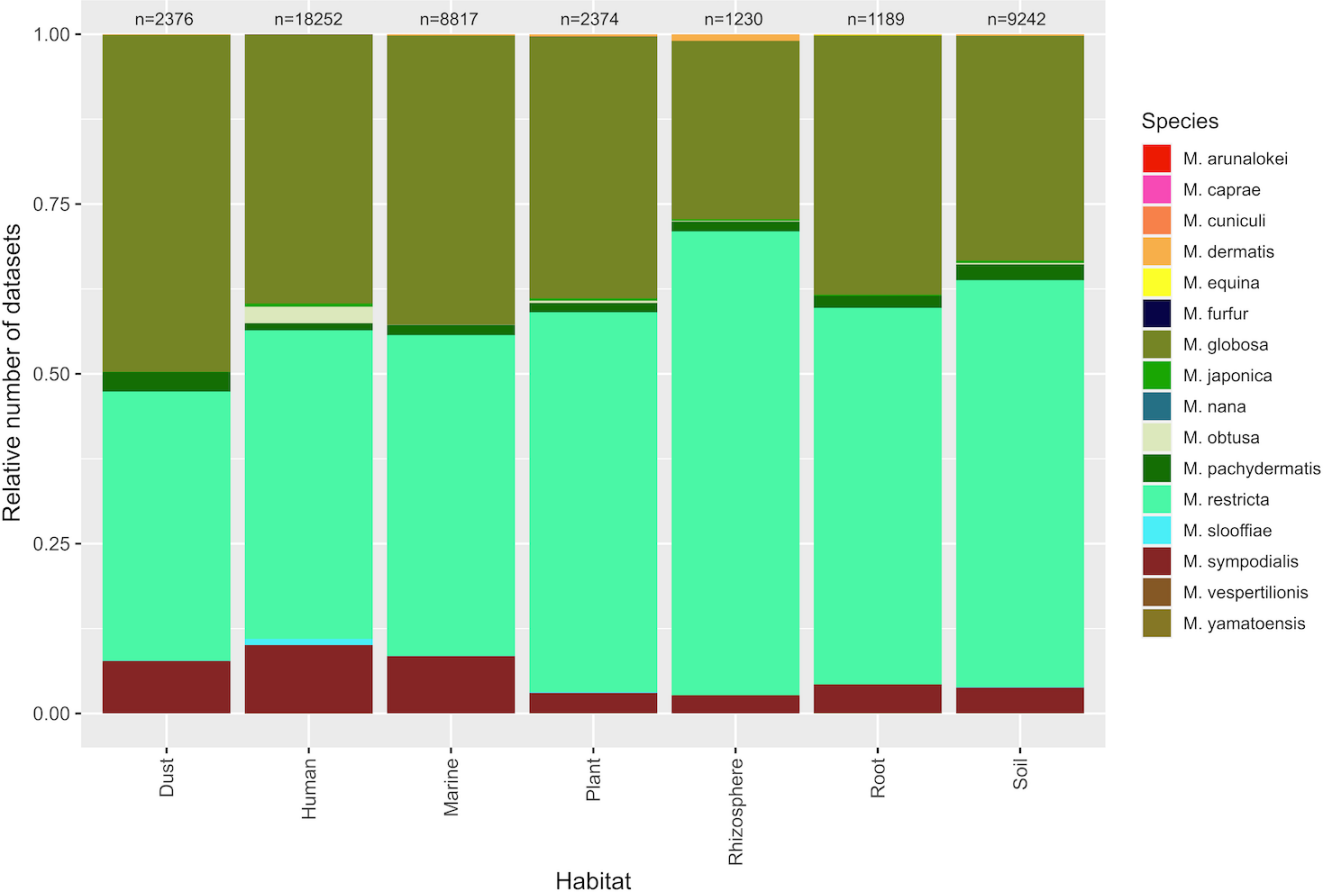
The relative occurrence of *Malassezia* species identified in different metagenomic data sets across various habitats was computed using the BigQuery platform.

BigQuery was employed to extract data on the prevalence of various *Malassezia* species across different environments. Our findings consistently showed that *M. restricta* and *M. globosa* were among the most prevalent species across all studied environments, followed by *M. sympodialis* and *M. pachydermatis* (Fig. 4). Similarly, in our investigation targeting ITS and MAT genes, our analysis revealed that *M. restricta* and *M. globosa* were the most abundant species, followed by *Malassezia arunalokei* both in amplicon and shotgun data (Fig. S2).

**Fig 5:**
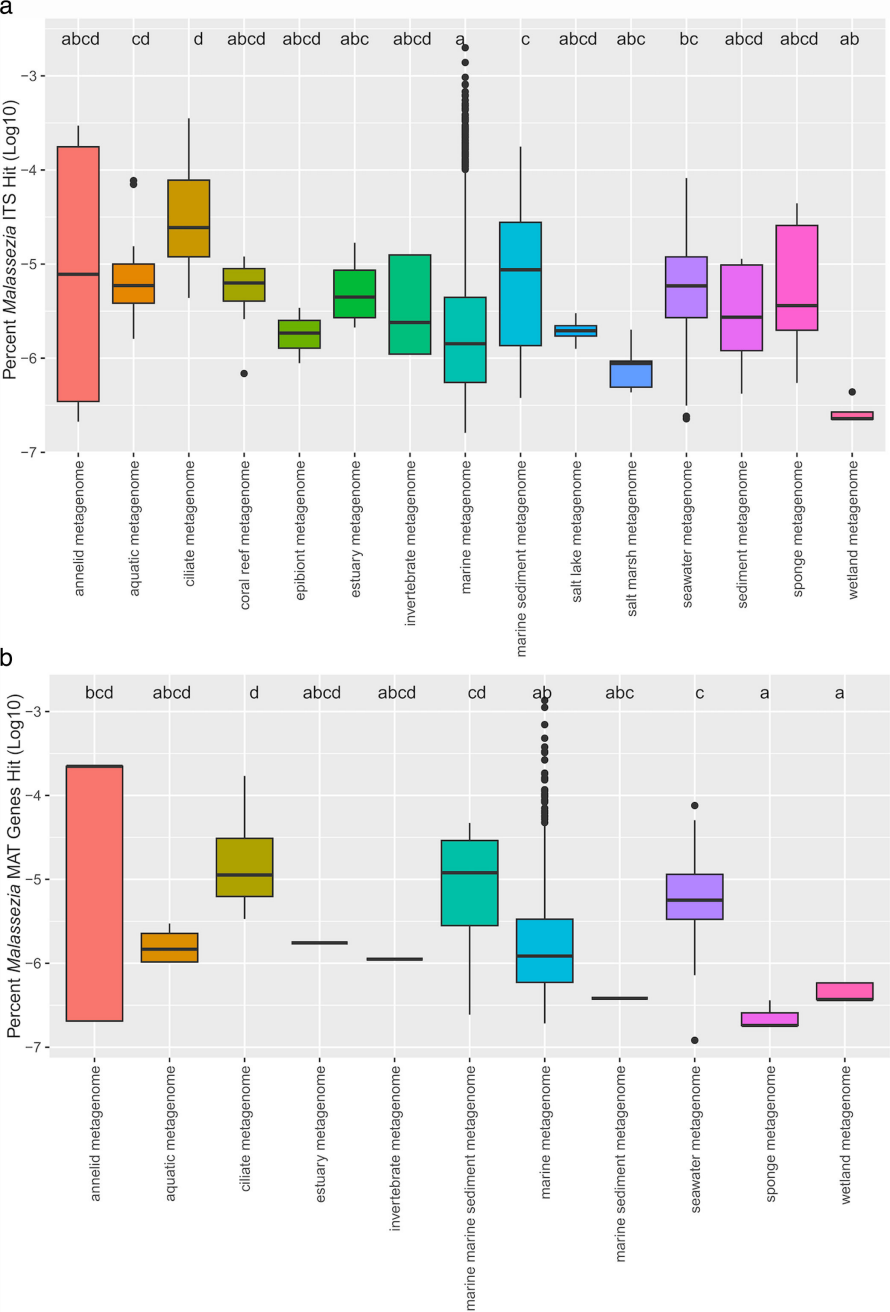
The number of combined *Malassezia* ITS1 and ITS2 sequences detected in various marine habitats from metagenomic data sets (a). A custom Perl script was used to search 11,510 metagenomic data sets from marine environments for the presence of *Malassezia* ITS sequences. Different letters denote significant differences in the number of *Malassezia* ITS hits among various habitat types at *P* < 0.05. The number of *Malassezia* mating type genes detected in various marine habitats (b). A custom Perl script was employed to search 11,510 metagenomic data sets from marine environments for the presence of *Malassezia* mating-type genes. Different letters denote significant differences in the number of *Malassezia* mating-type genes across habitat types at *P* < 0.05. Some data sets labeled as marine metagenomes in the SRA database do not clearly indicate whether they refer to water samples or other marine sources.

We then looked to identify marine data sets with the highest number of *Malassezia* reads that could be used for assembling a metagenome-assembled genome of *Malassezia*. Using four different approaches, scanning ITS sequences and MAT genes, PebbleScout, and BigQuery, we effectively pinpointed 11 data sets with the highest *Malassezia* reads and four data sets with the highest fungal reads overall from the SRA data (Table 1; Table S2). By analyzing 11,510 SRA data sets from marine environments, we found that 1,230 data sets contained hits (sequence match) to one of the 16 *Malassezia* species ITS sequences, while 352 data sets had hits to the MAT genes. In our investigation targeting ITS and MAT genes in marine shotgun data, we found that *Malassezia* hits were relatively low in abundance (with a maximum of 126 hits for ITS1 and ITS2 combined and 37 hits for MAT genes) compared to the significantly higher number of hits observed in human skin surface metagenomes (e.g., 4,700 ITS hits in SRR3644400). Similarly, in amplicon data from marine samples, we observed a maximum of 895 ITS hits in the marine sediment metagenome (SRR8467923), contrasting with the 139,124 hits detected in human gut metagenome data (ERR1424815). We did not find a significant variation in the number of *Malassezia* hits across different habitats (Fig. 5). We conducted a comparison between the number of ITS hits and BigQuery hits on shotgun data sets from marine environments, revealing a strong correlation (*P* < 3.17e-70, *R*_Pearson_ = 0.57) between the two methods, indicating their relative effectiveness (Fig. S3). Although each method has its limitations, we encountered zero ITS hits in SRA accessions DRR173070 and DRR173093, likely due to their smaller data set sizes (3–5 million reads). However, using BigQuery, we identified a high percentage of *Malassezia* reads within these data sets.

**TABLE 1:** Data sets enriched in *Malassezia* or fungi have been assembled, and *Malassezia* contigs have been extracted^*c*^

Accession	Sampling habitat	Assembly size	Total scaffolds	*N*_50_	*Malassezia* scaffolds	*Malassezia* assembly size	Human scaffolds	Total count *Malassezia* (%)^*a*^	Total count Hominidae (%)^*a*^	Malassezia ITS hits^*b*^	Malassezia MAT hits^*b*^	Total count fungi (%)^*a*^	BUSCO (%)
DRR173070	Marine	49,284,883	145,819	327	5,147	1,994,796	147	168,216 (10.707)	788,315 (50.177)	0	0	168,216 (10.707)	2.2
ERR538184	Marine	899,977,327	1,670,096	533	903	351,179	106	6,994 (0.003)	1,495,369 (0.730)	142	102	19,522 (0.010)	0.3
DRR173093	Marine	166,136,739	457,922	359	686	259,565	772	8,060 (0.302)	355,786 (13.327)	0	0	8,060 (0.302)	0.1
SRR14574967	Seawater	835,077,705	1,252,953	675	322	130,189	338	18,590 (0.027)	1,519,038 (2.182)	74	12	26,153 (0.038)	0
SRR12068625	Marine sediment	141,237,949	184,162	858	111	40,578	486	27,566 (0.078)	3,926,294 (11.052)	232	7	32,665 (0.092)	0
ERR4691748	Marine	1,239,091,400	2,098,953	572	64	24,843	11	6,184 (0.003)	208,846 (0.101)	37	8	11,188 (0.005)	0
SRR12068624	Marine sediment	142,164,795	185,955	933	60	22,957	136	33,857 (0.086)	3,512,400 (8.956)	100	67	80,966 (0.206)	0.1
ERR868506	Marine	711,311,015	1,168,303	597	47	17,116	6	1,672 (0.001)	141,623 (0.093)	65	31	211,839 (0.139)	0
SRR14574968	Seawater	720,307,614	1,031,744	729	45	17,169	188	6,652 (0.013)	875,760 (1.687)	24	2	9,251 (0.018)	0
ERR868443	Marine	1,025,499,140	1,651,322	614	43	14,607	12	1,102 (0.001)	148,293 (0.083)	41	4	185,847 (0.104)	0
SRR14574980	Seawater	562,796,973	742,765	856	42	15,748	109	8,085 (0.016)	689,618 (1.337)	38	39	14,613 (0.028)	0

^*a*^ Calculated using BigQuery (Google Cloud Platform).

^*b*^ Calculated using custom Perl script.

^*c*^ Assembly statistics and the number of hits for *Malassezia*, fungi, and Hominidae are provided.

**Fig 6:**
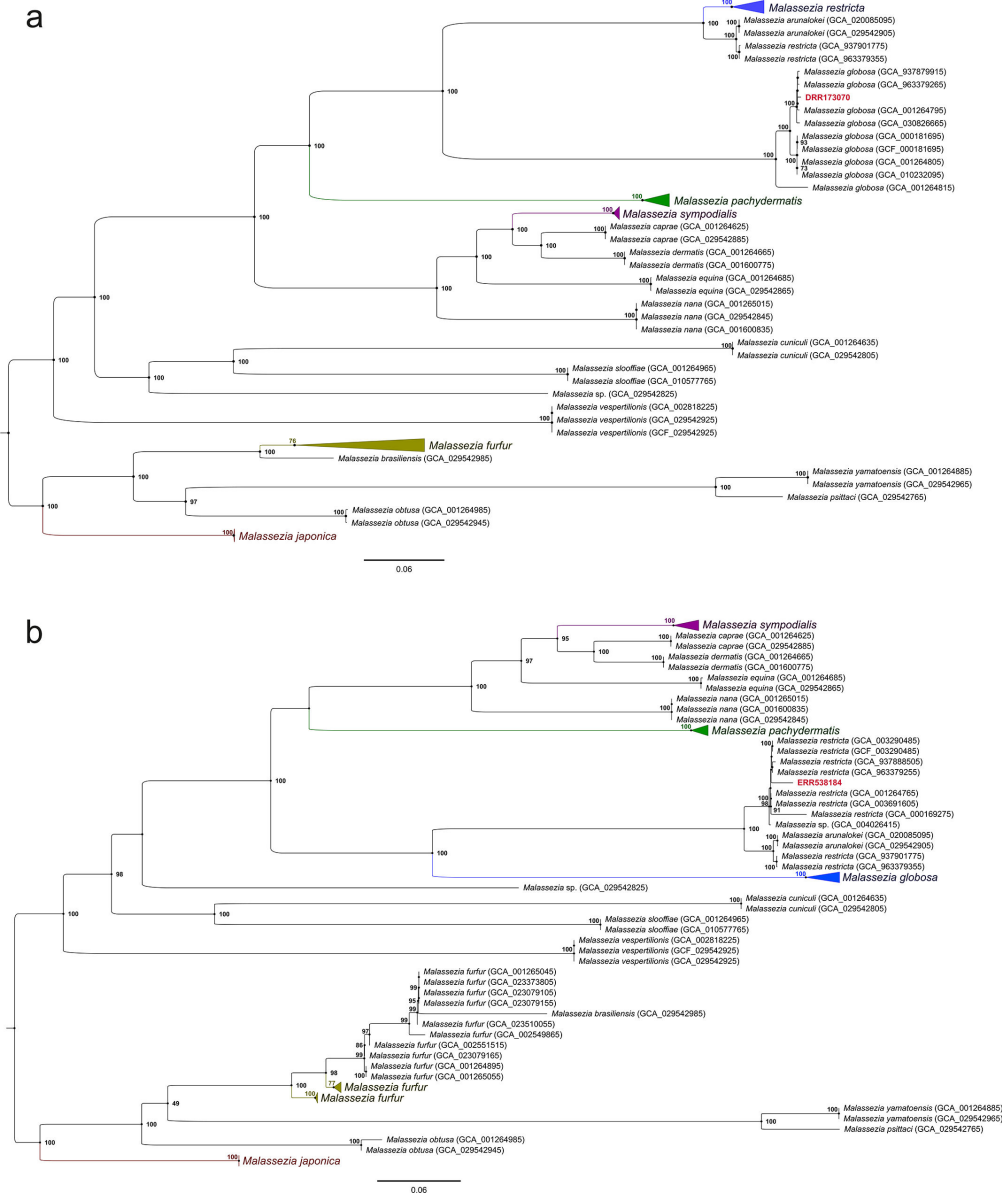
Phylogenetic tree constructed using 154 *Malassezia* genes extracted from the SRA data set number DRR173070 (a). Phylogenetic tree was constructed using 60 *Malassezia* genes extracted from the SRA metagenomic data set number ERR538184 (b). Contigs resolved as *Malassezia* from the data sets were mapped to 85 existing *Malassezia* assemblies, and the matched sequences were extracted from genomes, aligned, and concatenated using a custom Python script. The concatenated data set contained 25,860 and 6,937 character states, respectively. The tree was constructed with a maximum-likelihood approach using IQtree version 2.2.0.3 with 100 bootstrap replications.

Following this selection process, we proceeded with the assembly and purification of *Malassezia* contigs from these data sets. We assembled and binned a total of 38 data sets from marine environments and could successfully identify bins labeled as *Malassezia* in just four data sets (Table 1; Table S2). The analysis revealed low genome completeness levels of *Malassezia* bins, with a maximum of 2.2%, across the data sets examined. Initial examination identified small numbers of genes for *Malassezia* bins, including 154, 60, 18, and 8 within the first four data sets. Phylogenetic trees were constructed using these genes for all corresponding *Malassezia* species, indicating that the assembly derived from our most *Malassezia*-rich data set (DRR173070) corresponded to *M. globosa*, while the remaining assemblies were attributed to *M. restricta* (Fig. 6; Fig. S4 and S5). Notably, both *M. globosa* and *M. restricta* are among the most prevalent species found on human skin. The alignment of *Malassezia* contigs retrieved from the SRA data sets DRR173070 and ERR538184 with existing genomes of *M. globosa* and *M. restricta* shows significant similarity across multiple regions of the genome, as illustrated in Fig. S6 and S7. We did not detect any *Malassezia* bin in the data set with higher fungal abundance. However, in this study, we successfully identified metagenome-assembled genomes (MAGs) for several fungal species, such as *Setomelanomma holmii*, *Cadophora* sp., and *Exophiala xenobiotica*, achieving high genome completeness (>98% BUSCO completeness) using the MAG-Explorer pipeline. We also detected and assembled a *Saccharomyces* bin of higher completeness (16.4%) and used this assembly to suggest that it was introduced from human-associated sources (Fig. S8). We detected varying degrees of positive correlation between the occurrence of hits for different pairs of *Malassezia* species: *M. restricta* and *M. globosa*, *M. restricta* and *M. arunalokei*, *Malassezia caprae* and *Malassezia dermatis*, *Malassezia equina* and *M. caprae*, *M. equina* and *M. dermatis*, and *M. sympodialis* and *M. restricta* (see Fig. S9 and S10). This observation is likely attributable to the high sequence identity in their ITS regions. Their phylogenetic affinity confirms this relationship (Fig. S4 and S5).

The *Tara* Ocean expedition is one of the largest studies of marine biodiversity, encompassing ecosystems from polar regions to tropical waters. We explored *Tara* Oceans amplicon and shotgun data for the protist size fraction for studying *Malassezia* occurrence. We hypothesized that shotgun and amplicon data generated for the same samples should indicate a strong correlation for *Malassezia* abundance if either the environmental sample had *Malassezia* in it or if *Malassezia* was introduced during the sampling procedure. Alternatively, if there is no correlation between amplicon and metagenomic estimates of *Malassezia* abundance, then it would argue that *Malassezia* was introduced after DNA extraction. We looked for the sequencing run accessions associated with both the amplicon and shotgun data from the same sample and used BigQuery to compute the *Malassezia* hits for both the amplicon and shotgun data. An analysis of the Pearson correlation coefficient reveals a robust correlation (*P* < 0.05) between the amplicon and shotgun data for *Malassezia* sequence hits (Fig. S11). To validate the findings derived from the BigQuery search, we obtained a list of sample IDs for which sequences were clustered into OTUs (W5 database) and ensured that shotgun data for these samples were available. We found 10 *Malassezia* OTUs and calculated the total number of *Malassezia* sequences for each sample from the results of reference 51. The analysis showed a strong correlation (*P* < 0.05) between the number of sequences grouped within *Malassezia* OTUs and the count of *Malassezia* hits in the shotgun and amplicon data (Fig. S12 and S13). These data are consistent with either *Malassezia* being present in the original water sample or with the introduction of *Malassezia* during the sampling process.

**Fig 7:**
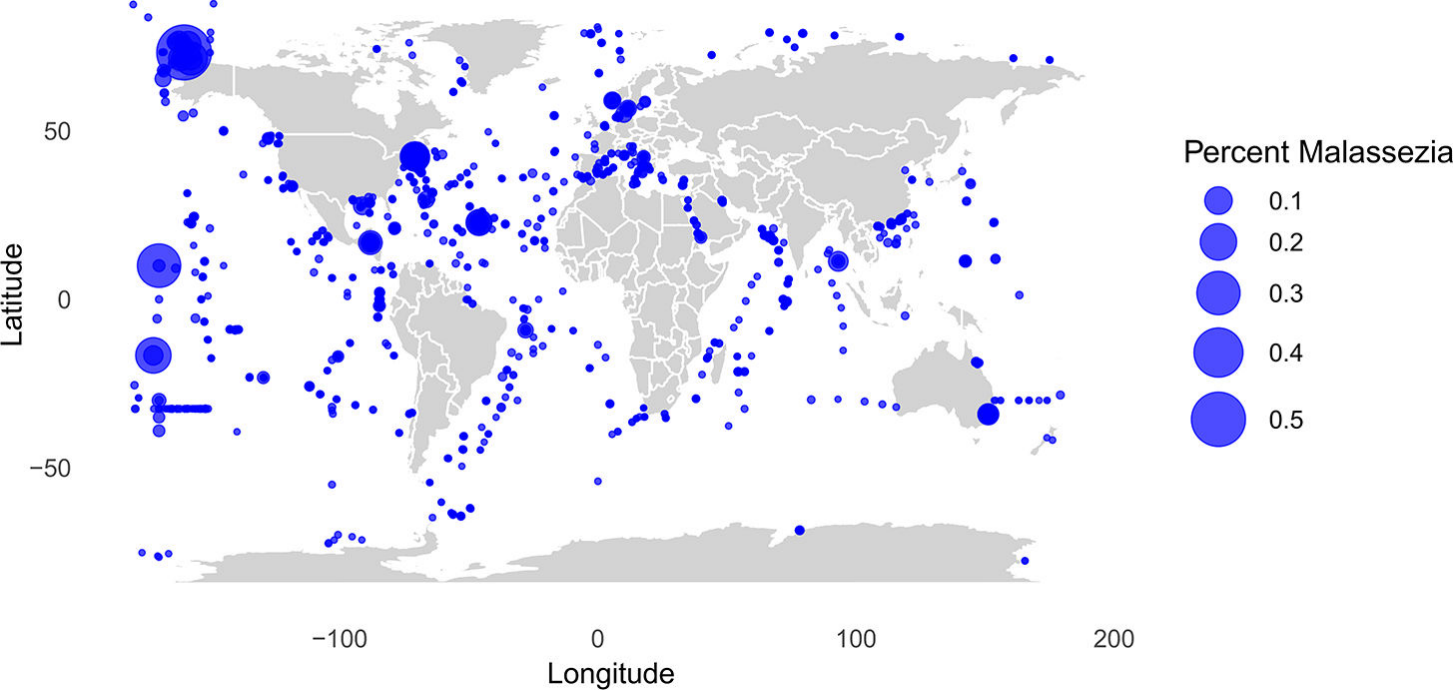
The world map illustrates the sampling locations. Each point marks a shotgun sequenced sample, and the size of the circle reflects the percentage of *Malassezia* sequences identified in that sample.

We hypothesized that human activities in coastal areas could lead to the contamination of marine environments by *Malassezia* prior to sampling. Specifically, we hypothesized that *Malassezia* may disperse from land into the water as a consequence of these activities, where it could potentially persist in marine environments and be detected via DNA sequencing. To explore this hypothesis, we calculated the distance to the nearest shore for each sample and examined the correlation between *Malassezia* abundance and distance to land. Our results showed a significant, although weak, trend that sampling locations closer to land contain a greater proportional abundance of *Malassezia* than those farther offshore (*R*_Pearson_ = −0.18, *P* = 0.9.14e-16; Fig. 7; Fig. S14).

## DISCUSSION

The presence of *Malassezia* in marine samples can be explained by at least four non-exclusive scenarios: (i) the organisms may be native to the ocean, reproducing and subsisting on marine hosts and substrates, (ii) they could regularly disperse from terrestrial habitats, possibly persisting in marine environments, (iii) their presence might result from human contamination during sampling, with the expectation that they do not reproduce or persist in marine habitats, or (iv) *Malassezia* may not be found in the ocean at all but instead could be introduced during laboratory processes like PCR and library preparation. The evidence presented highlights several patterns suggesting both human-associated and environmental influences on the microbial community (Table 2). The predominance of human-associated *Malassezia* species and their strong correlation with human DNA strongly suggest a human-driven origin, supporting the hypothesis of anthropogenic contamination. In contrast, the correlation between other saprobes and human DNA is less definitive, with some indications that these species may also be influenced by human activity, though with more uncertainty. The WGS/amplicon correlation data, however, supports consistent species identification across different methodologies and suggests pre-extraction contamination is more likely. The correlation with distance to shore suggests that these microbial communities may be affected by land-based dispersal, but the weakness of the correlation suggests it may be more influenced by other factors, possibly linked to human mishandling of the samples or contamination after sampling in the lab. The prevalence of *Malassezia* in non-anthropogenic habitats raises the possibility that these species are connected to human contamination or inputs from other mammals inhabiting these environments, though the latter remains uncertain. On the other hand, the discovery of novel *Malassezia* lineages and the presence of species in hydrothermal/subsurface environments (9, 10) further complicates the scenario, suggesting that some species may belong to specialized ecosystems that are likely unrelated to human contamination. The generally low abundance of *Malassezia* across samples suggests an environmental presence or contamination rather than an autochthonous presence. Together, these pieces of evidence imply a complex interplay of human and environmental factors that require careful interpretation to separate true ecological signals from contamination biases.

**TABLE 2:** Overview of evidence supporting four possible scenarios for *Malassezia* distribution^*a*^

Evidence	Autochthonous		Disperse from land		Sampling Contamination		Lab contamination	
	Consistent with	Inconsistent with	Consistent with	Inconsistent with	Consistent with	Inconsistent with	Consistent with	Inconsistent with
Mostly human-associated *Malassezia* species		√	√		√		√	
Correlation with human DNA		√	√		√		√	
WGS/amplicon correlation	√		√		√			√
Correlation with distance to shore	√		√		√		√	
Similar prevalence in other non-anthropogenic habitats		√		√	√		√	
Novel species diversity	√			√		√		√
Presence in hydrothermal/subsurface	√			√		√	√	
No difference in low biomass/high biomass samples	√		?	?		√		√
Generally low abundance		√	√		√		√	

^*a*^ “√” = evidence in support of input source; “?” = evidence equivocal.

We reasoned that we should be able to assemble a metagenome-assembled genome of a marine-inhabiting *Malassezia* from at least one metagenome data set out of over 11,000 available, given that perhaps one of these samples would “catch” *Malassezia* locally thriving. Instead, we found a widespread (>2,000 data sets) occurrence of *Malassezia* across marine data sets but at low relative abundance, almost exclusively present when human DNA was present, and we were only able to bin *Malassezia* contigs from four samples. Our analysis revealed that *M. restricta* and *M. globosa* were the most frequently encountered species detected within the environmental data sets. These species were consistently present across various samples, which is consistent with the presence of *Malassezia* as a contaminant in many studies. Moreover, the four bins were most closely associated with human-associated genomes (i.e., clinical samples) of *M. restricta* and *M. globosa*. The high prevalence of these particular *Malassezia* species suggests that they might be common contaminants introduced during sample collection or processing, possibly due to their ubiquity on human skin.

*Malassezia* was detected across all tested terrestrial habitats using various sequencing approaches. Here, we determined that dust and human samples exhibited a higher abundance of *Malassezia* reads than other habitats (Fig. 3). This observation is consistent with expectations, as humans are recognized as a primary reservoir of *Malassezia*. It is likely that dust samples were predominantly collected from environments inhabited by humans, where human skin and hair are predominant. Moreover, the findings suggest the potential for widespread introduction of *Malassezia* by humans into diverse habitats, given *Malassezia*’s presence as a commensal organism on human skin. The occurrence of *Malassezia* in dust could explain the significant correlation of *Malassezia* abundance and distance from land if explained by allochthonous inputs of *Malassezia* from terrestrial to marine environments. However, the weakness of this correlation may indicate a greater likelihood that *Malassezia* is introduced during sampling or laboratory processes in most samples in which it occurs.

Humans are not the only hosts for *Malassezia*; this genus is also associated with the skin of various mammals worldwide, including domestic animals like dogs and cats. For instance, *M. pachydermatis* colonizes the skin of dogs and can lead to skin infections such as otitis externa (52), while *Malassezia equina* is frequently found on the skin, mane, and tail of horses (53). Research has also documented the occurrence of *Malassezia* in wild animals, with various species identified on birds like pigeons and parrots (54, 55), as well as in wild mammals such as bats. Interestingly, *Malassezia* has been found in the guts of marine mammals, including dolphins and whales (56), and invertebrates such as snails and lobsters (57), although no diseases caused by *Malassezia* have been reported in marine mammals. Given the widespread distribution of mammals, including humans, it is likely that their skin and hair can shed into soil, water, and other environments, where they may be collected along with environmental samples.

Since microbes associated with humans and other animals can contaminate environmental samples, their presence in environmental data sets raises important questions about their ecological roles and behaviors. Certain microbes, such as *Malassezia*, are predominantly linked to warm-blooded animals, whereas others, like *Saccharomyces*, thrive in a greater breadth of ecological settings. We observed weak positive correlations between human DNA and widespread saprotrophic fungi, as well as other eukaryotes, across marine samples. These correlations may be explained by shared genetic sequences due to common evolutionary ancestry, where conserved genetic markers and biological pathways across eukaryotic species could lead to erroneous assignment of reads when two related lineages are present in a sample. Specifically, these correlations could be artifacts of bioinformatics methods, particularly errors in taxonomic assignment during large-scale genomic queries, where sequence misidentifications or biases may create spurious detection of co-occurrence between organisms. Further analysis is needed to clarify whether these correlations are biologically meaningful or computational artifacts. It remains uncertain whether the widespread presence of *Malassezia* in environmental samples signifies the fungus’ ability to persist independently of its typical hosts, such as in pelagic marine environments. Given *Malassezia*’s presumed inability to synthesize fatty acids, if it is found in marine environments, it is likely to be in a symbiotic relationship with other organisms. However, the detection of *Malassezia* transcripts in various studies (26–28) leads to the open question of whether RNA extractions are contaminated by human sources or if the transcripts originate from genuinely active organisms. One possibility is that *Malassezia* can persist and even grow in certain circumstances in a marine environment, regardless of its origin.

Contamination from laboratory reagents also poses a significant challenge in environmental studies, particularly for researchers working with low-biomass microbial samples. The presence of contaminating DNA can compromise the accuracy and reliability of results, affecting both rRNA gene sequencing projects, which rely on targeted PCR amplification, and shotgun metagenomic studies, which do not. Contamination has been well documented for bacteria (18, 58, 59) and fungi (19), highlighting the widespread impact of this issue. Laboratory reagents such as water, extraction kits, chemicals, and consumables can introduce exogenous microbial DNA into environmental samples, leading to misleading conclusions about microbial diversity and community composition. Additionally, background contamination from airborne particles, laboratory surfaces, and even researchers’ skin and clothing further complicates efforts to ensure data integrity. This pervasive issue underscores the need for stringent contamination controls and careful experimental design to minimize its impact on environmental microbiome studies.

A significant limitation of this study is that we can only identify *Malassezia* species that closely match those already described and genome sequenced. If novel *Malassezia* species are present in marine ecosystems, they may go undetected, as both genomic and ITS rDNA reads could be challenging to classify. Moreover, considering the presence of novel diversity in marine habitats (10), it is conceivable that two types of *Malassezia* might exist in these environments: one that infects humans and is widely distributed, and another that could be confined to marine habitats. However, this distinction is speculative and requires further research to validate the presence and ecological roles of these potential types.

### Conclusion

*Malassezia* was detected across nearly all tested terrestrial and marine habitats, using various assay methods. Phylogenetic analysis identified *M. globosa* and *M. restricta* as the most prevalent species in marine environments. Dust samples, likely collected from human living environments, exhibited a significantly higher abundance of *Malassezia* reads. We observed a significant correlation between the number of human and *Malassezia* sequences in all studied habitats. These findings suggest widespread co-introduction of *Malassezia* and human DNA into samples. After identifying marine metagenomes with the highest proportion of *Malassezia* reads, metagenome assembly identified bins corresponding to *Malassezia*, although they exhibited low genome completeness. Despite this limitation, our findings revealed that these microbial sequences shared notable similarities with species typically associated with humans. Similar methods with other fungal-rich samples yielded nearly complete genome assemblies of other fungi, suggesting the methods we employed were robust. Future research is crucial to identify and understand how to identify common microbial contaminants that obscure the accurate characterization of indigenous microbial communities. This is essential for ensuring the reliability and accuracy of environmental microbial analyses.

## Data Availability

The MAG-Explorer pipeline and scripts for mining the SRA database and the pipeline for constructing phylogenies from partial genomes can be found in the GitHub repository (https://github.com/Rahimlou).

## SUPPLEMENTAL MATERIAL

Supplemental Material mbio.01142-25-s0001.pdf Figures S1-S14; supplemental text.

ASM does not own the copyrights to Supplemental Material that may be linked to, or accessed through, an article. The authors have granted ASM a non-exclusive, world-wide license to publish the Supplemental Material files. Please contact the corresponding author directly for reuse.
