# Supplementary material for: Malassezia in environmental studies is derived from human inputs
Source: mBio. 2025 May 19;16(6):e01142-25. doi: 10.1128/mbio.01142-25 (PMC12153345; doi:10.1128/mbio.01142-25)
Supplement: Supplemental Material — Figures S1-S14; supplemental text. [file mbio.01142-25-s0001.pdf]

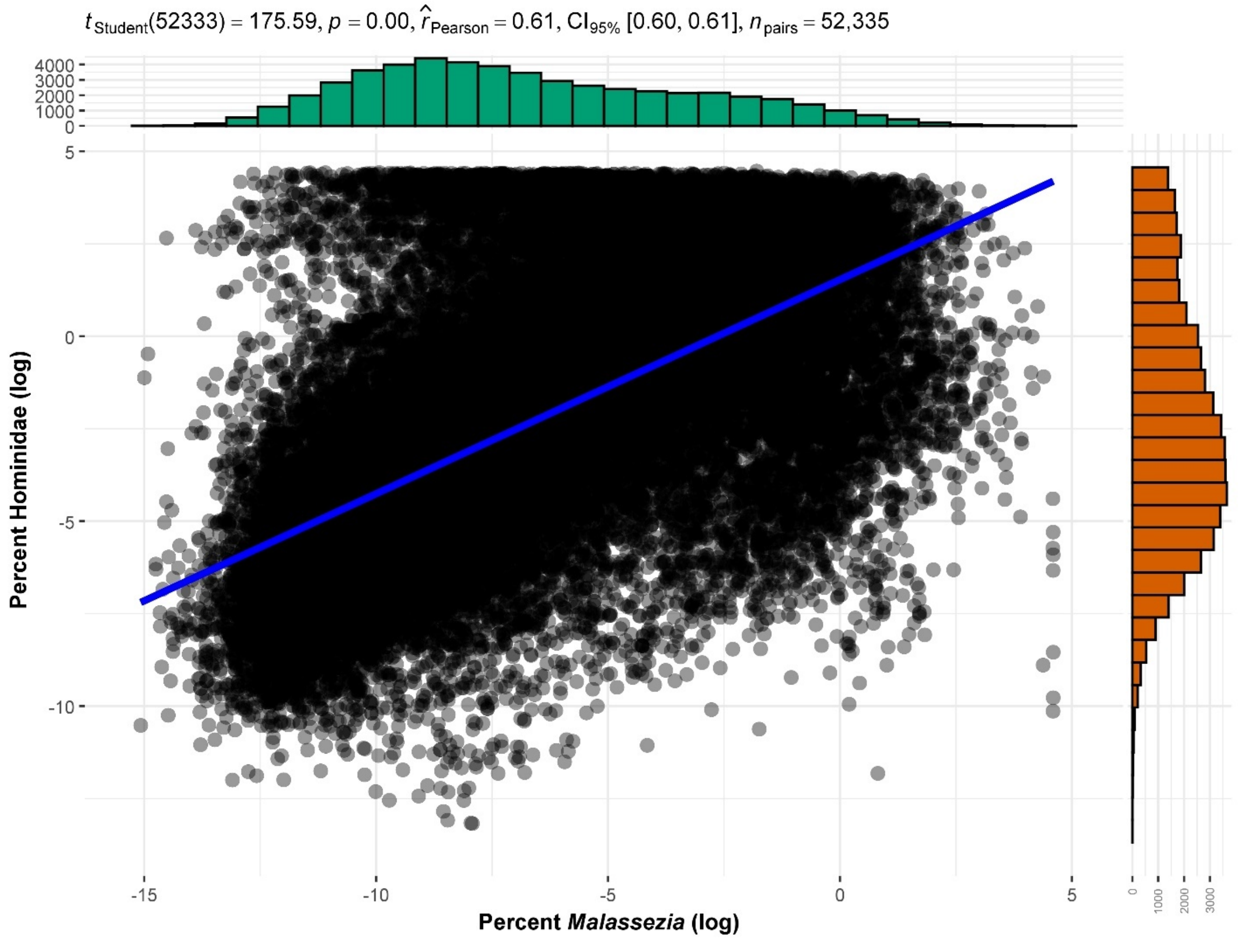

Figure S1. Correlation between the number of log-transformed human (Hominidae) and *Malassezia* hits within all shotgun data from various habitats (except human) extracted using Big Query.

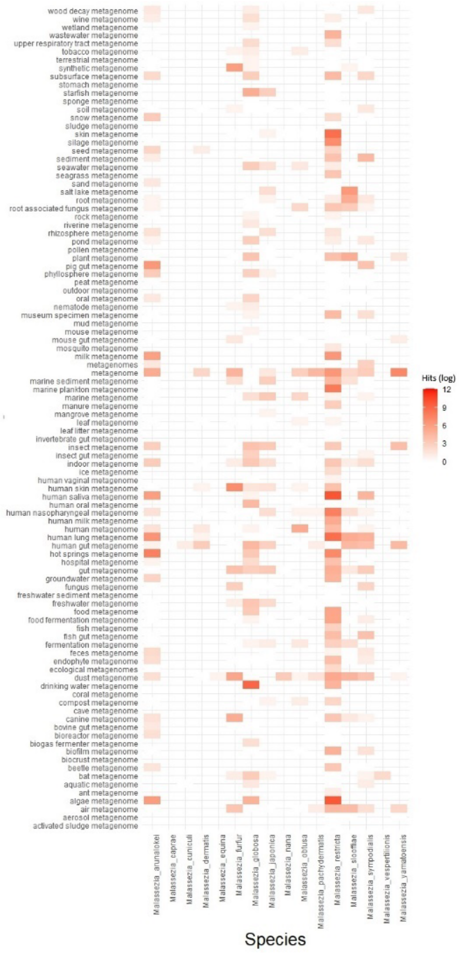

Figure S2. The relative occurrence of *Malassezia* species identified in different amplicon datasets across various habitats was calculated using custom Perl script.

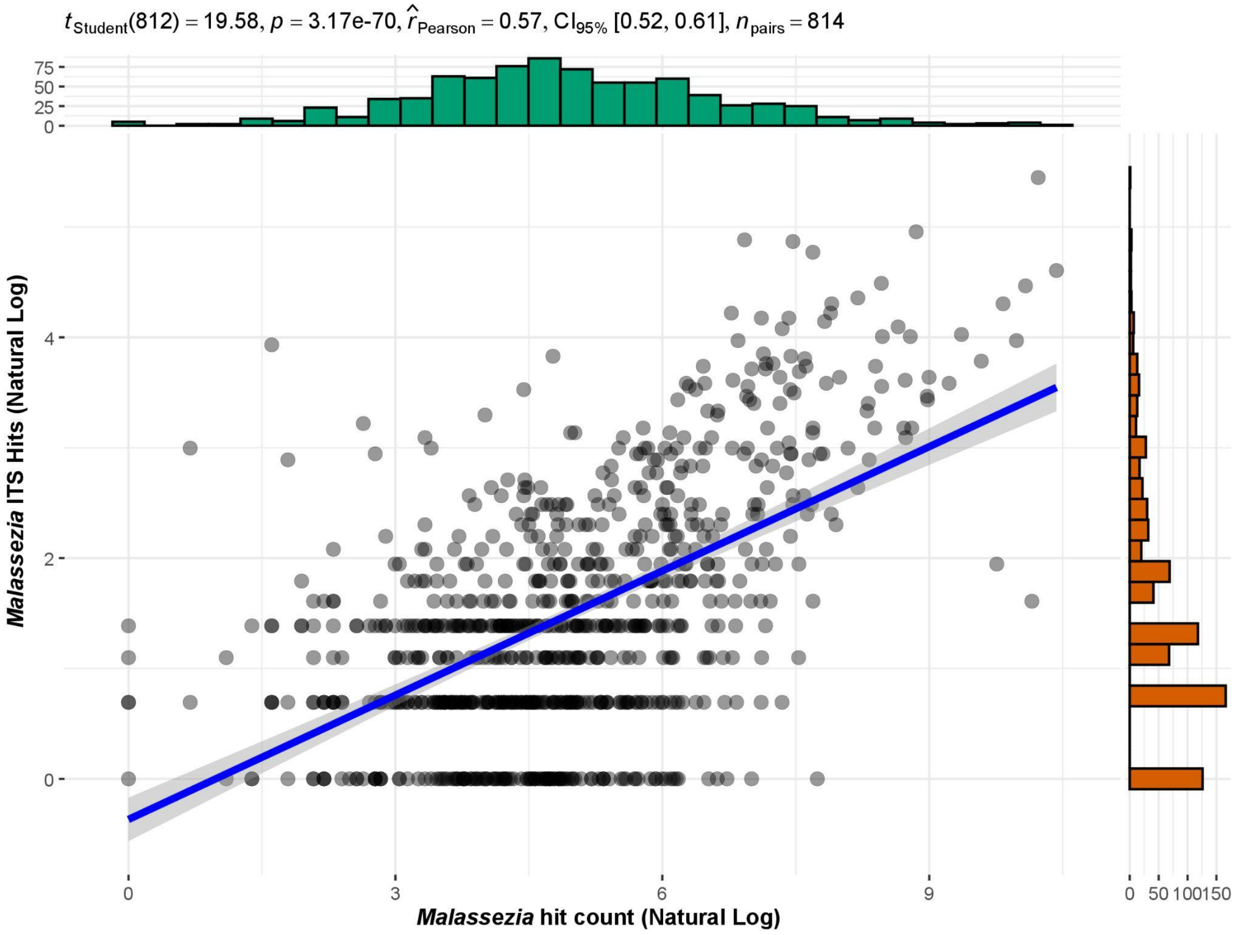

Figure S3. Correlation between the number of *Malassezia* ITS sequence hits using our pipeline and *Malassezia* hits using the Big Query platform on metagenomic data from marine environments.

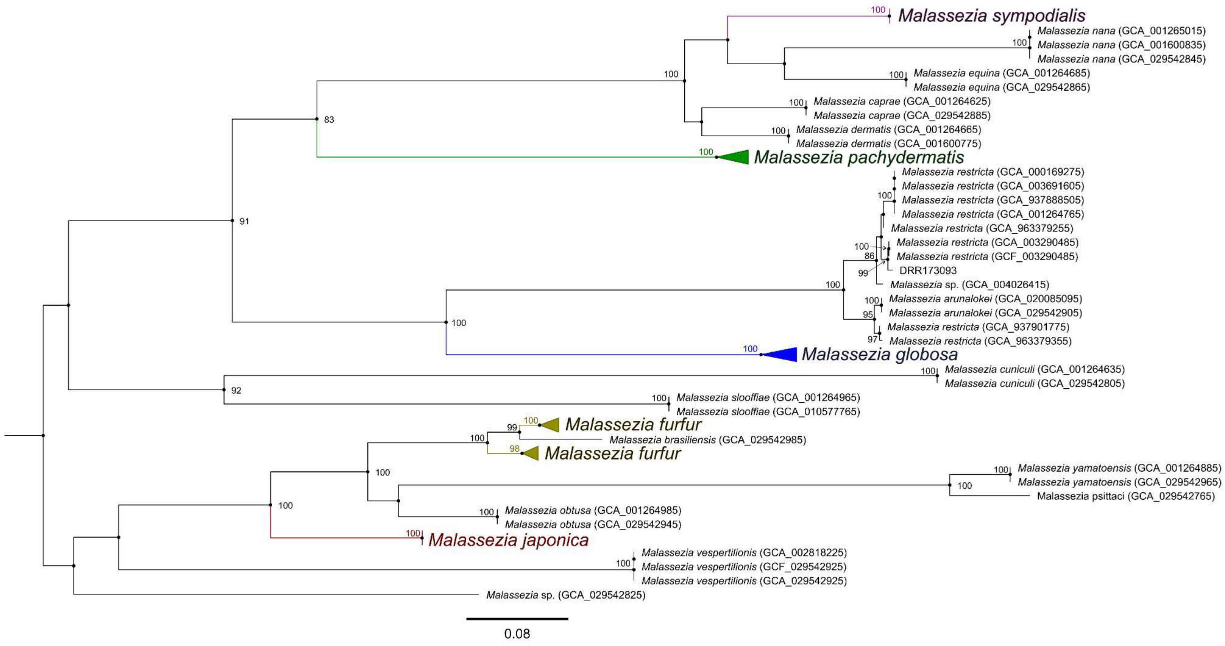

Figure S4. Phylogenetic tree constructed using eight *Malassezia* genes extracted from the SRA dataset number DRR173093. Contigs resolved as *Malassezia* from the datasets were mapped to 85 existing *Malassezia* assemblies and the matched sequences were extracted from genomes, aligned, and concatenated using a custom Python script. The tree was constructed with a maximum-likelihood approach using IQtree v. 2.2.0.3 with 100 bootstrap replications.

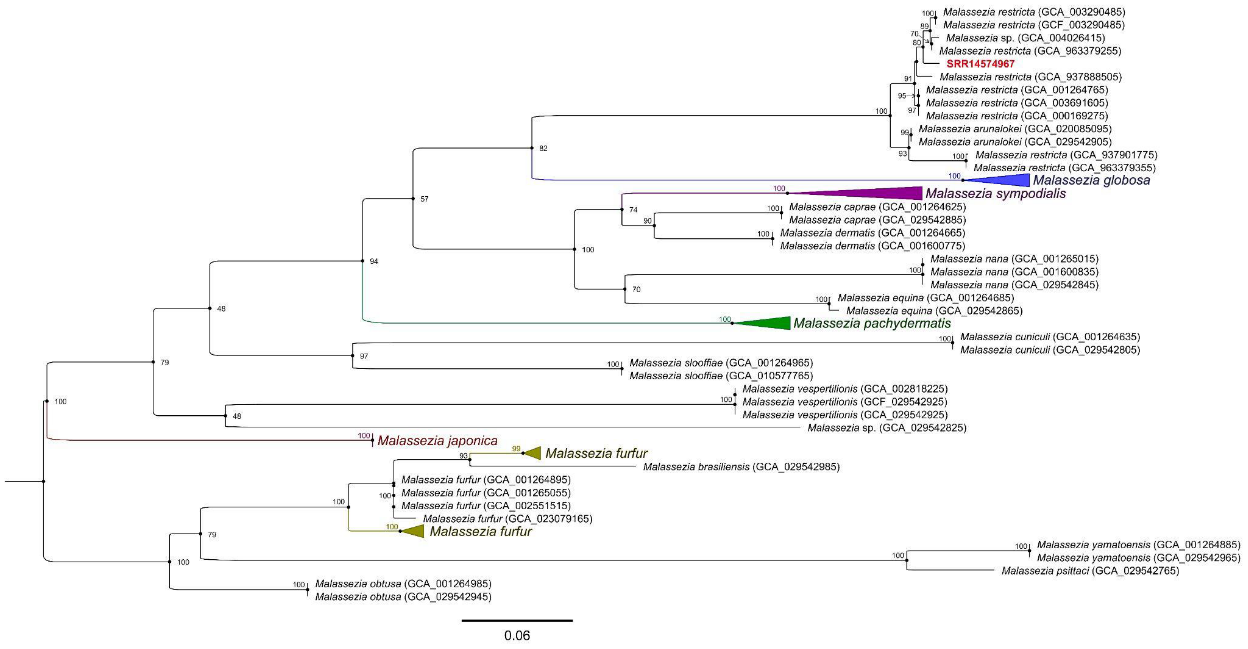

Figure S5. Phylogenetic tree constructed using 18 *Malassezia* genes extracted from the SRA dataset number SRR14574967. Contigs resolved as *Malassezia* from the datasets were mapped to 85 existing *Malassezia* assemblies and the matched sequences were extracted from genomes, aligned, and concatenated using a custom Python script. The tree was constructed with a maximum-likelihood approach using IQtree v. 2.2.0.3 with 100 bootstrap replications.

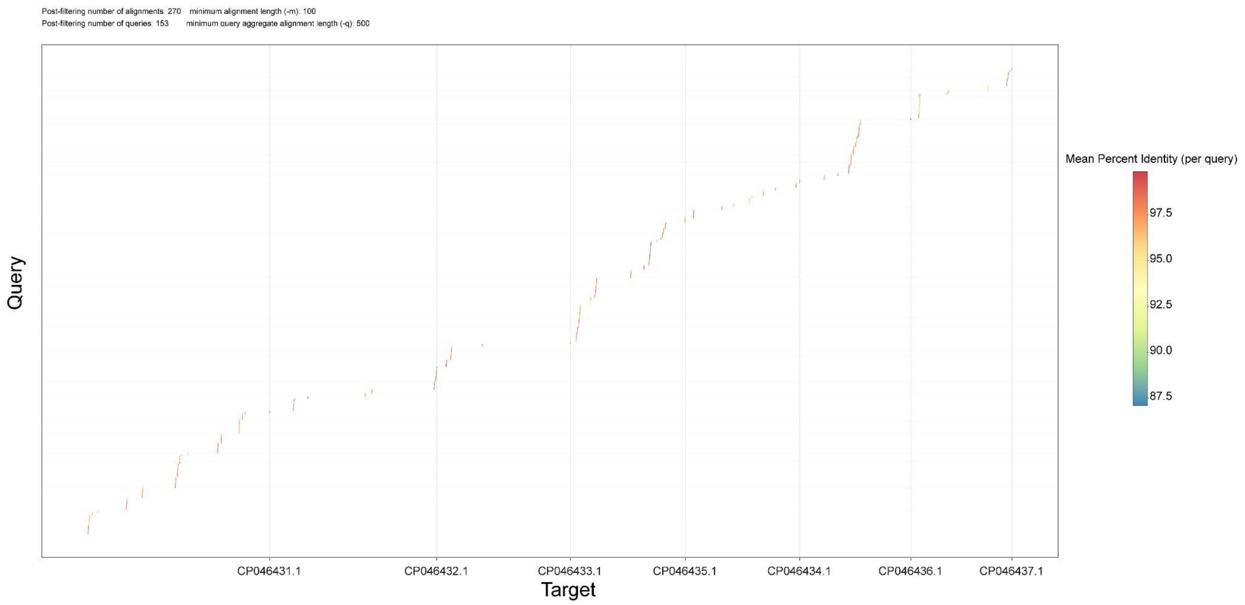

Figure S6. *Malassezia* contigs recovered from DRR173070 dataset aligned to *M. globosa* genome (GCA_000181695) using MUMmer v. 4.0.0.

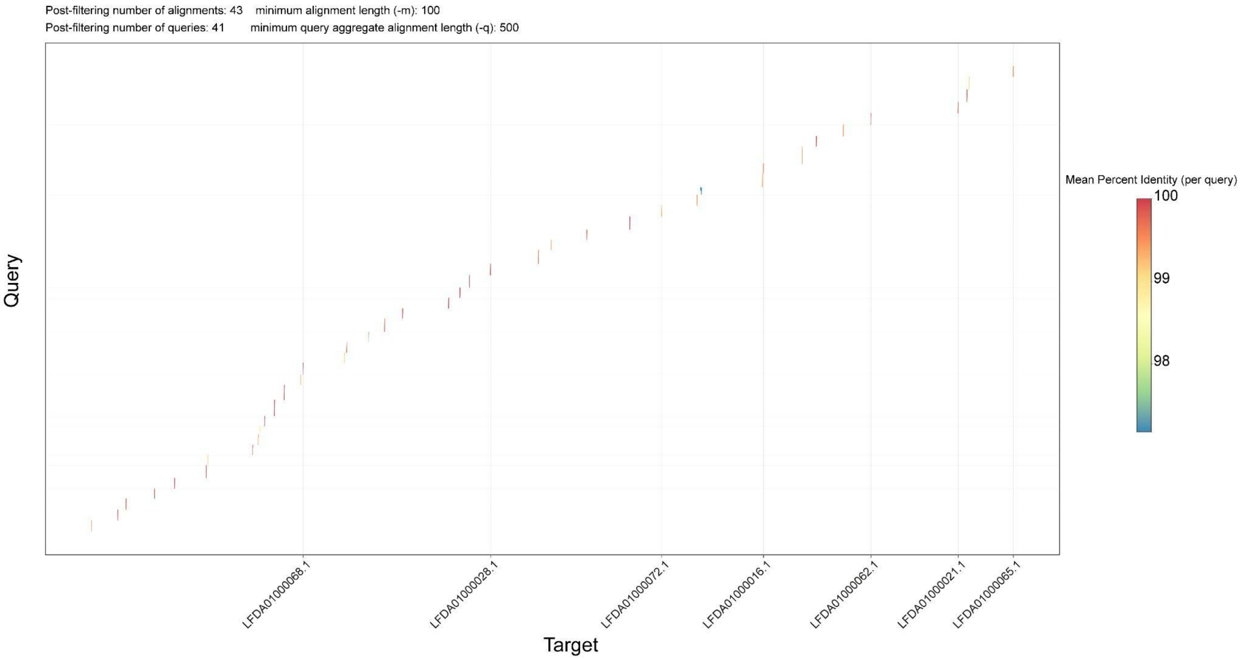

Figure S7. *Malassezia* contigs recovered from ERR538184 dataset aligned to *M. restricta* genome (GCA_003290485) using MUMmer v. 4.0.0.

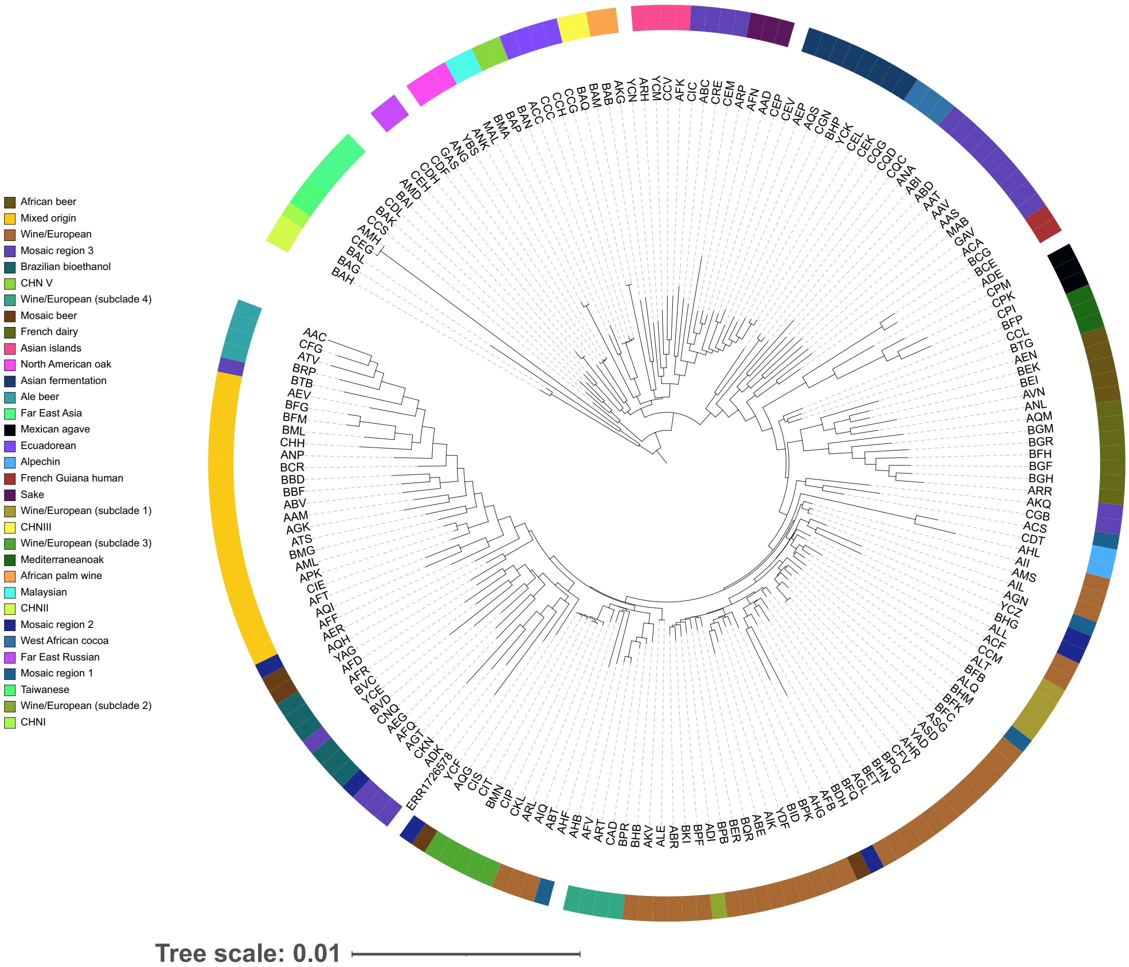

Figure S8. The phylogenetic tree of *Saccharomyces cerevisiae* was constructed using 2,675 genes and 2,315,356 character states. The genomes included are a subset of Peter et al. (2018), encompassing all resolved clades from that study. The tree was generated using a maximum-likelihood approach with IQ-TREE v2.2.0.3, utilizing the Model Finder Plus (MFP) option to select the best-fitting substitution model.

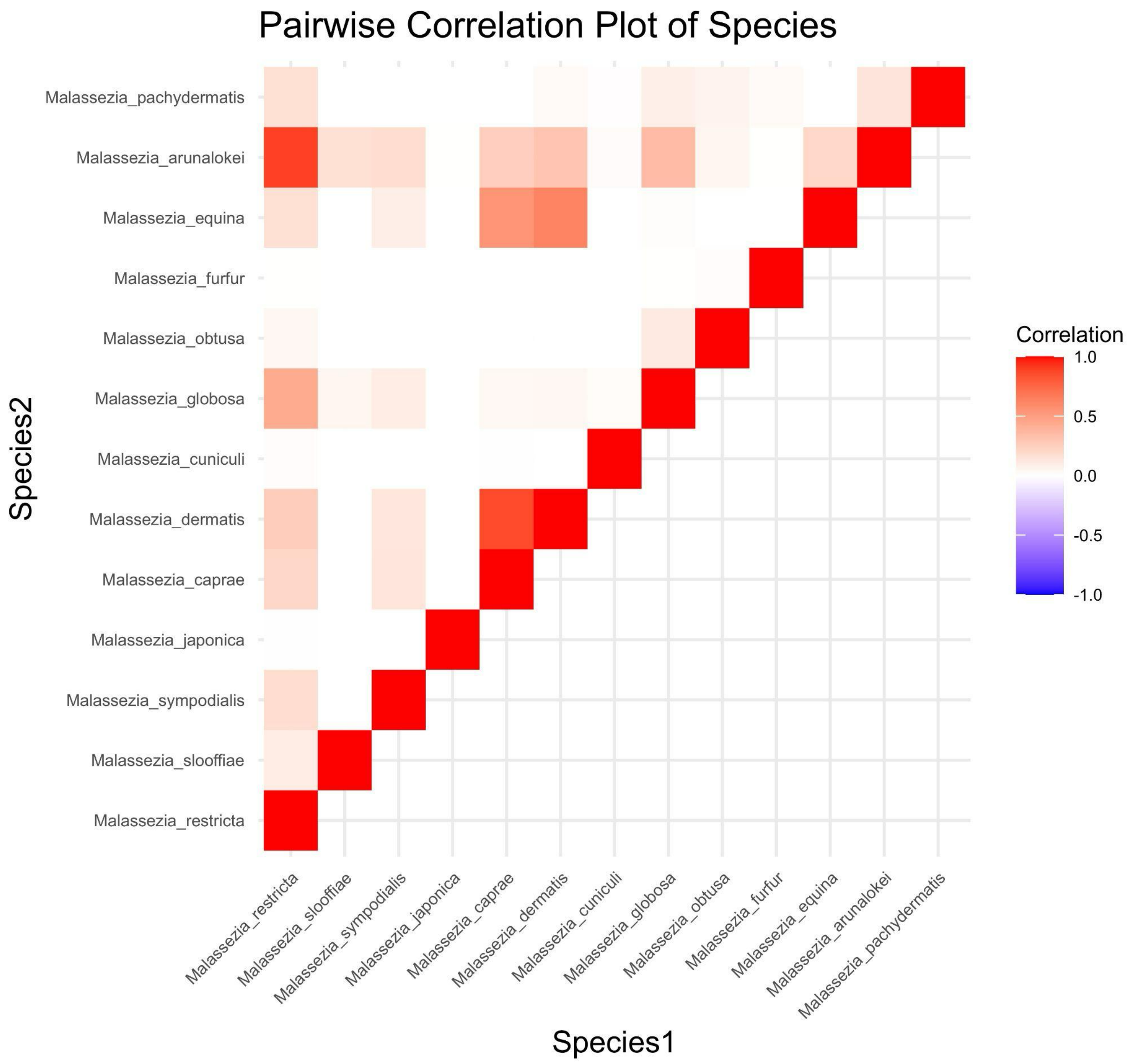

Figure S9. Correlation among *Malassezia* species based on the number of ITS hits in shotgun data from marine environments.

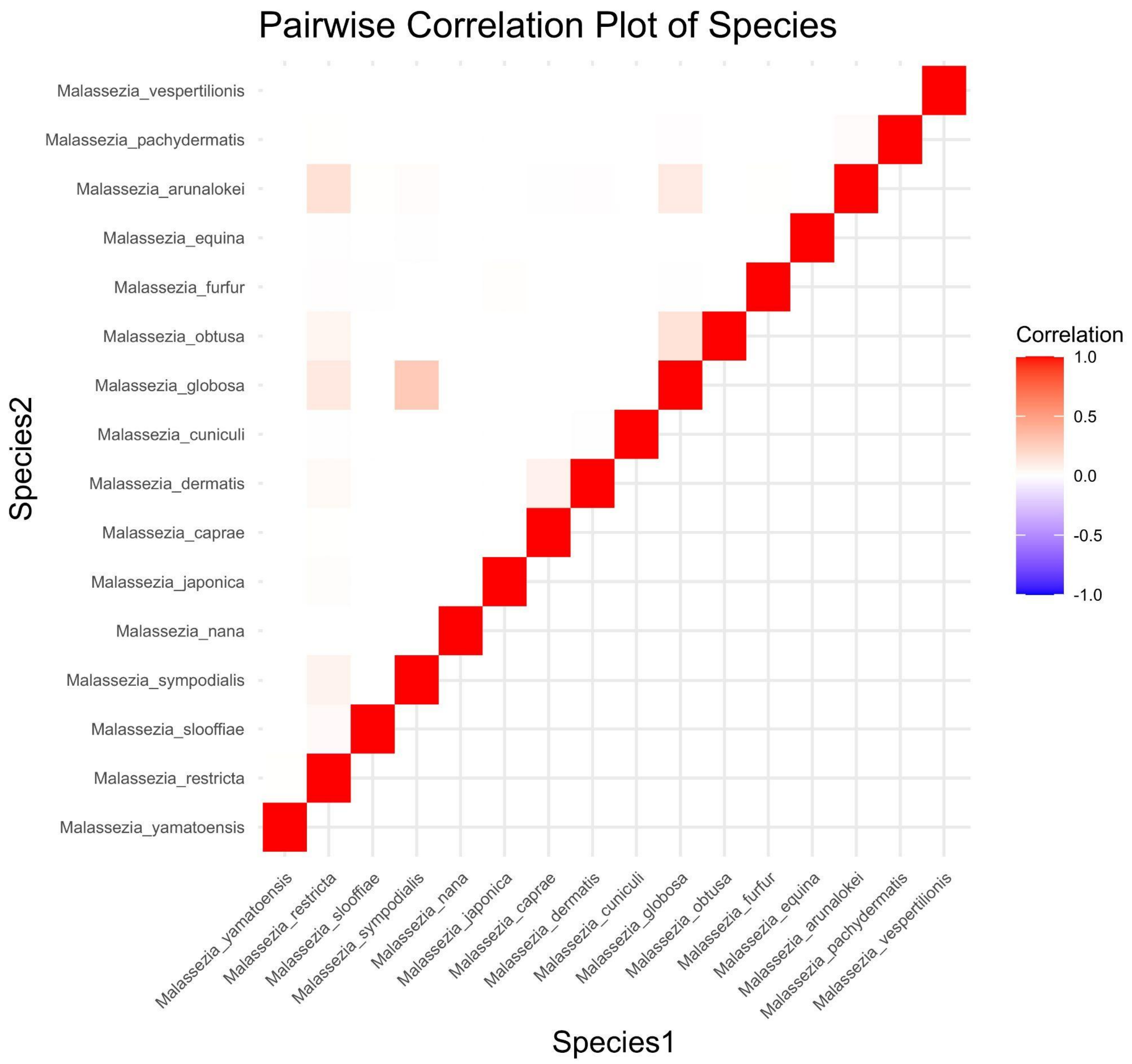

Figure S10. Correlation among *Malassezia* species based on the number of ITS hits in amplicon data from various environments.

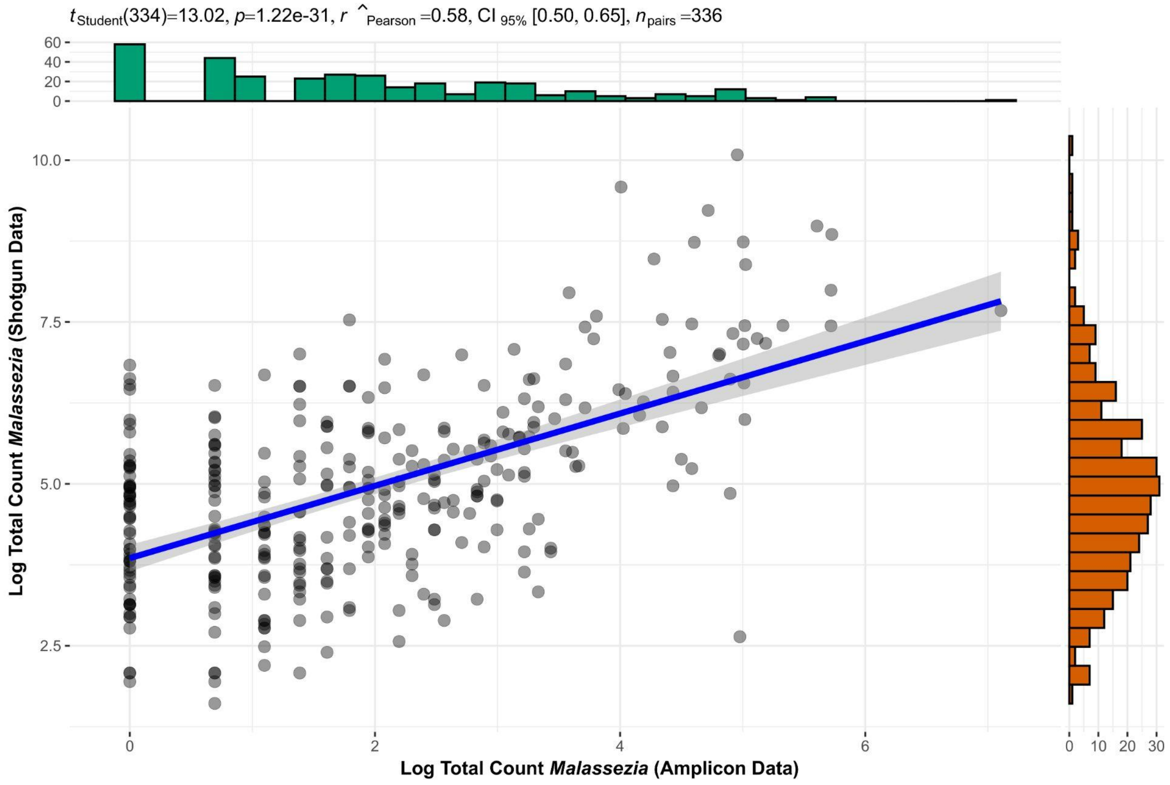

Figure S11. Correlation between the natural log transformed count of *Malassezia* hits in shared samples sequenced either through amplicon or shotgun methods in the *Tara* Ocean study. *Malassezia* hits for each dataset obtained using the Big Query platform.

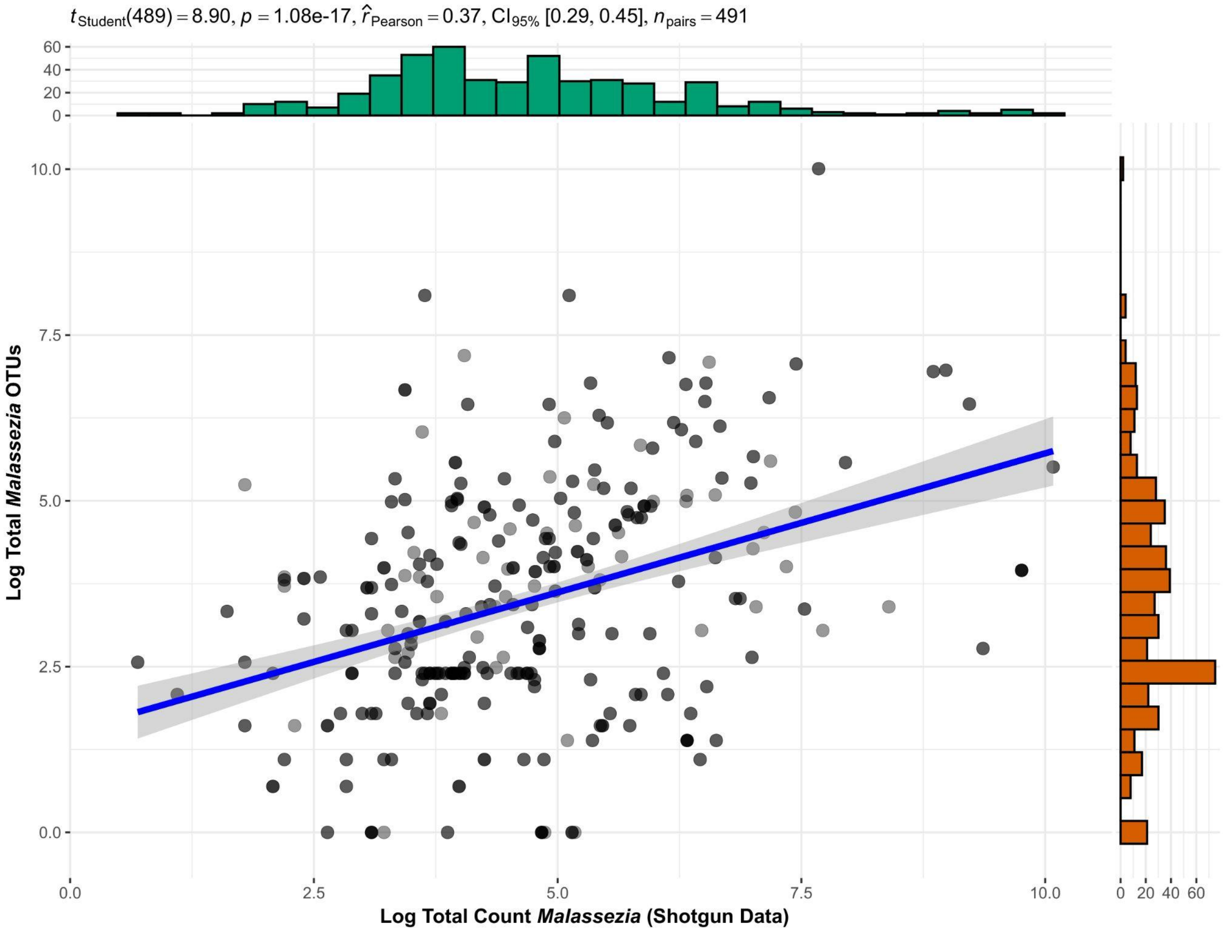

Figure S12. Correlation between the natural log-transformed count of Malassezia hits in shared samples sequenced via the shotgun method and the number of sequences clustered into Malassezia OTUs in the Tara Ocean study. Malassezia hits for shotgun data were obtained using the Big Query platform, while Malassezia OTUs were derived from the W5 database of the Tara Ocean study.

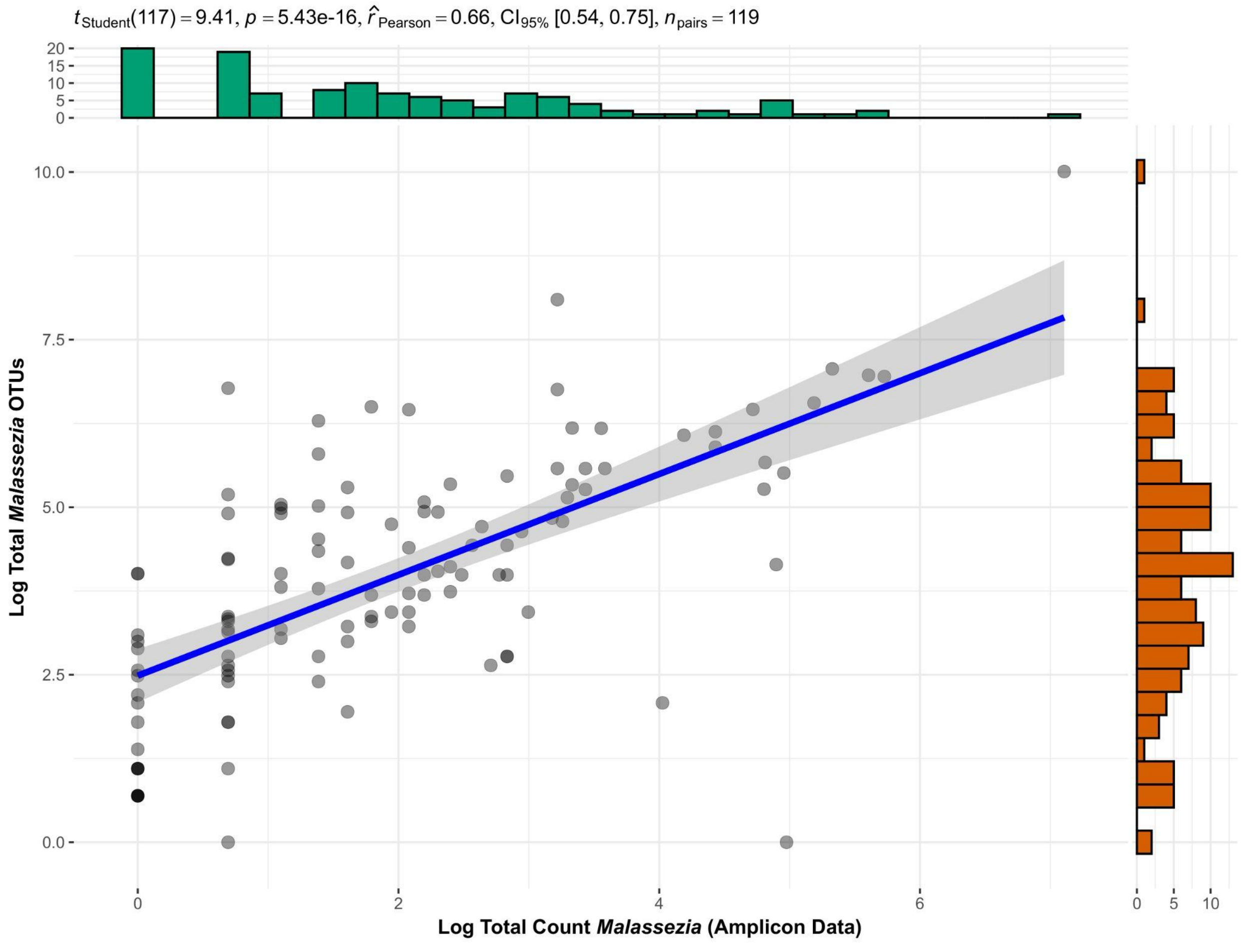

Figure S13. Correlation between the natural log-transformed count of *Malassezia* hits in shared samples sequenced via the amplicon method and the number of sequences clustered into *Malassezia* OTUs in the Tara Ocean study. *Malassezia* hits for amplicon data were obtained using the Big Query platform, while *Malassezia* OTUs were derived from the W5 database of the Tara Ocean study.

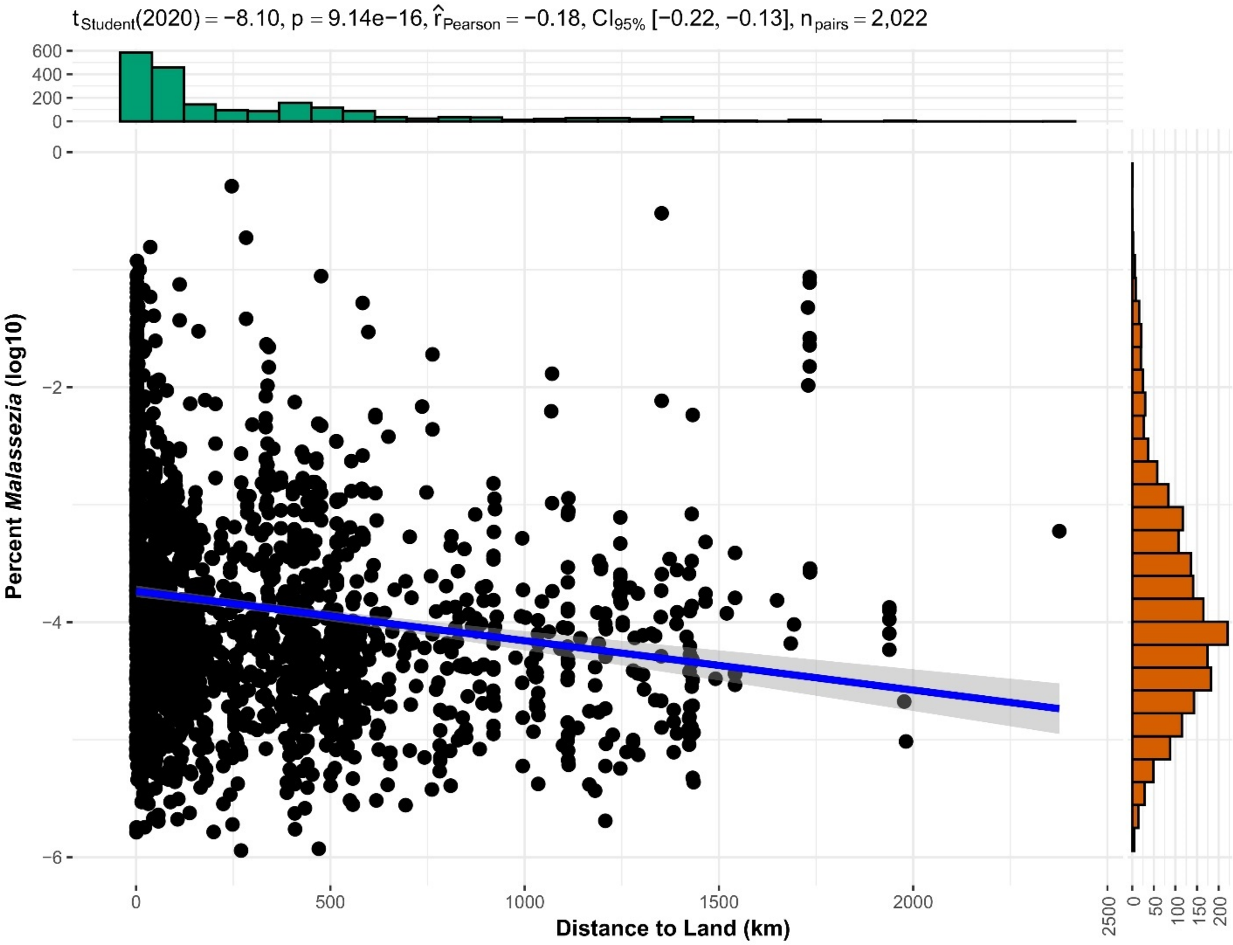

Figure S14. Correlation between the abundance of *Malassezia* in shotgun sequenced biosamples and the proximity of these biosamples to land.

Supplementary Text

We analyzed shotgun data from marine environments for the presence of *Saccharomyces* as another human mycobiota member and identified several datasets containing abundant *Saccharomyces* sequences. We then assembled the genomes from these datasets and constructed a phylogenetic tree using the same method used for *Malassezia* incorporating 186 additional *Saccharomyces* genomes from various resolved *Saccharomyces* clades, subsetted from the Peter et al. (2018) dataset. For constructing the phylogeny, we employed our custom pipeline, which is specifically tailored for constructing phylogenies from partial genomes.

Since *Saccharomyces* is a frequent component of the human mycobiota, and is also prevalent in environmental samples, we anticipated that its distribution would be similar to that of *Malassezia*. Given *Saccharomyces'* widespread presence in various human-associated habitats, it is likely that co-introduction of both *Saccharomyces* and human could occur, analogous to the widespread presence of *Malassezia*. Understanding the extent and impact of human-associated microbes in the oceans is crucial for accurate microbial ecology studies by distinguishing between true environmental presence and human-derived contamination. To explore this, we have assembled *Saccharomyces* sequences from marine environments. Our aim was to construct a phylogenetic tree to discern whether we could trace a marine *Saccharomyces* to a distinct lineage given the large number of genome sequences of strains available. Our phylogenetic analysis revealed that the partial genome of *Saccharomyces* cerevisiae recovered from ERR1726578 (16.4% BUSCO genome completeness) clustered with strains from mosaic region 3 (Fig. S14). Peter et al. (2018) identified three groups of mosaic strains, concluding that these strains are predominantly associated with human-related environments. This implies that a comparable situation may be occurring with *Saccharomyces*, where contamination could have resulted from mishandling of the sample and contact with human skin.
